# Relaxing parametric assumptions for non-linear Mendelian randomization using a doubly-ranked stratification method

**DOI:** 10.1371/journal.pgen.1010823

**Published:** 2023-06-30

**Authors:** Haodong Tian, Amy M. Mason, Cunhao Liu, Stephen Burgess

**Affiliations:** 1 MRC Biostatistics Unit, School of Clinical Medicine, University of Cambridge, Cambridge, United Kingdom; 2 British Heart Foundation Cardiovascular Epidemiology Unit, Department of Public Health and Primary Care, University of Cambridge, Cambridge, United Kingdom; University Hospital of the Canton Vaud (CHUV), SWITZERLAND

## Abstract

Non-linear Mendelian randomization is an extension to standard Mendelian randomization to explore the shape of the causal relationship between an exposure and outcome using an instrumental variable. A stratification approach to non-linear Mendelian randomization divides the population into strata and calculates separate instrumental variable estimates in each stratum. However, the standard implementation of stratification, referred to as the residual method, relies on strong parametric assumptions of linearity and homogeneity between the instrument and the exposure to form the strata. If these stratification assumptions are violated, the instrumental variable assumptions may be violated in the strata even if they are satisfied in the population, resulting in misleading estimates. We propose a new stratification method, referred to as the doubly-ranked method, that does not require strict parametric assumptions to create strata with different average levels of the exposure such that the instrumental variable assumptions are satisfied within the strata. Our simulation study indicates that the doubly-ranked method can obtain unbiased stratum-specific estimates and appropriate coverage rates even when the effect of the instrument on the exposure is non-linear or heterogeneous. Moreover, it can also provide unbiased estimates when the exposure is coarsened (that is, rounded, binned into categories, or truncated), a scenario that is common in applied practice and leads to substantial bias in the residual method. We applied the proposed doubly-ranked method to investigate the effect of alcohol intake on systolic blood pressure, and found evidence of a positive effect of alcohol intake, particularly at higher levels of alcohol consumption.

## Author summary

Various exposures, such as alcohol consumption, may exhibit different effect sizes on health outcomes at different exposure levels. Studying the non-linear shape of these effects can provide valuable insights to predict the impact of interventions for different individuals. Mendelian randomization is an epidemiological approach to make causal inferences from observational data. It uses genetic variants to divide the population into subgroups which behave similarly to arms of a randomized trial. The current residual stratification method most often implemented for non-linear Mendelian randomization relies on strong parametric assumptions and can yield misleading results when these assumptions are violated. We propose a new stratification method that relaxes these assumptions. Our new doubly-ranked method demonstrates superior performance over the residual method across a wide range of simulation scenarios. Furthermore, in cases where the exposure is coarsened (for example, its value is rounded, say to the nearest whole number), the doubly-ranked method achieves good results while the residual method fails to handle such situations adequately. The doubly-ranked method can test the assumptions underlying the residual method, thereby assessing the validity of previously published results. We advocate for the doubly-ranked method to be used in preference to the residual stratification method for non-linear Mendelian randomization.

## Introduction

Mendelian randomization is an epidemiological technique that uses genetic variants as instrumental variables to make causal inferences from observational data [1, 2]. An extension to the method, known as stratified non-linear Mendelian randomization, first divides the population into strata with different average levels of the exposure, and then performs separate instrumental variable analyses in each stratum to obtain stratum-specific estimates, referred to as localized average causal effect (LACE) estimates [3, 4]. This allows researchers to explore the shape of the causal relationship between the exposure and the outcome. While other methods have been proposed for performing non-linear instrumental variable analysis [5–7], such methods typically require the parametric model relating the exposure to the outcome to be specified, or else perform model selection amongst a set of models [8]. Inferences from such approaches can be sensitive to the specification of the non-linear function or model selection procedure [9]. Additionally, it is difficult to fit detailed non-linear models if the instrumental variable takes a small number of discrete values, or explains a small proportion of the variance in the exposure; both of these scenarios are common in Mendelian randomization.

The stratification method for non-linear Mendelian randomization has been used to investigate the shape of the exposure–outcome relationship for body mass index (BMI) with mortality [10], systolic blood pressure with cardiovascular disease [11], and vitamin D with a range of outcomes [12].

**Fig 1:**
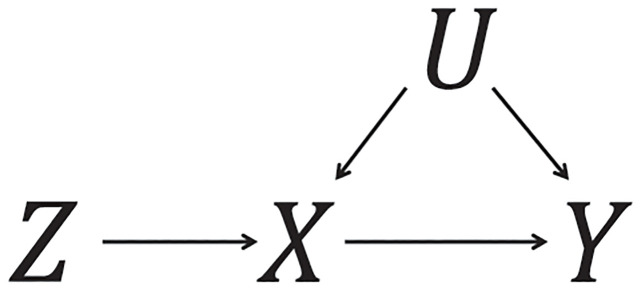
Directed acyclic graph (DAG) illustrating the instrumental variable assumptions. The exposure is denoted as *X*, the genetic instrument as *Z*, the outcome as *Y*, and exposure–outcome confounders as *U*. The exposure *X* is a collider in this DAG, as it is a common effect of the instrument and confounders.

An important technical detail in these analyses is that stratification is not performed on the exposure directly. The reason is that the exposure is a collider in the standard directed acyclic graph for an instrument variable: it is a common effect of the instrument and the exposure–outcome confounders (Fig 1) [13]. If we regard the instrumental variable as equivalent to random allocation in a randomized trial, the exposure is a post-randomization covariate, and so stratification on the exposure is inappropriate [14]. The original proposal for stratified non-linear Mendelian randomization was to first calculate a variable referred to as the “residual exposure” and stratify on this [3]. The residual exposure is calculated by regressing the exposure on the genetic instrument (either a single genetic variant or a score comprising multiple genetic variants), and taking the residual from this equation. By the properties of linear regression, the residual exposure is independent of the genetic instrument, and so strata defined using the residual exposure are independent of the genetic instrument. A weakness of this approach is that it relies on strong parametric assumptions of linearity and homogeneity between the genetic instrument and the exposure [15].

In this paper, we propose a new stratification method that does not require strict parametric assumptions to create strata of the population that have different average levels of the exposure, but are independent of the instrument. We demonstrate in a simulation study that the method, referred to as the doubly-ranked stratification method, can obtain unbiased LACE estimates when the effect of the instrument on the exposure is non-linear or heterogeneous. In contrast, LACE estimates from the residual stratification method are biased in these scenarios. We consider different types of the instrument and different confounder–outcome relationships. We also consider the scenario in which the exposure is coarsened (that is, either rounded or binned into categories), as is the case for several epidemiological risk factors. This scenario represents a difficulty for the residual stratification method, but can be accommodated by the doubly-ranked method. We apply the doubly-ranked method to study the shape of the causal effect of alcohol intake on systolic blood pressure (SBP). Software for implementing the doubly-ranked method is available in the DRMR package at https://github.com/HDTian/DRMR or as part of the SUMnlmr [16] package at https://github.com/amymariemason/SUMnlmr.

## Methods

### Ethics statement

The UK Biobank study has approval from the North West Multicentre Research Ethics Committee (11/NW/0382). Participants provided written consent to the use of their medical records and samples to be used for health-related research purposes: see https://www.ukbiobank.ac.uk/media/05ldg1ez/consent-form-uk-biobank.pdf for consent statement.

### Assumptions and data set-up

We assume the existence of a genetic instrument that satisfies the core instrumental variable assumptions [17, 18]:

(i) the instrument is associated with the exposure (relevance);
(ii) the instrument is not associated with the outcome via a confounding pathway (exchangeability);
(iii) the instrument does not affect the outcome directly, only possibly indirectly via the exposure (exclusion restriction).

For interpretability of the instrumental variable estimates, we additionally make either the monotonicity or homogeneity estimation assumption [19]. The monotonicity assumption states that the effect of the instrument on the exposure is non-negative for all individuals in the population (or alternatively, it is non-positive for all individuals in the population). There are various versions of the homogeneity assumption; the simplest version of the assumption states that the effect of the instrument on the exposure is constant for all individuals in the population [19, 20]. Under the monotonicity assumption, the instrumental variable estimate represents a local average treatment effect; under the homogeneity assumption, it represents an average treatment effect. The LACE is defined as the average causal effect for a subset of the population defined by stratification [3]. Depending on the estimation assumption, it either represents a local average treatment effect (monotonicity) or an average treatment effect (homogeneity) for that subset of the population. Note that here ‘localized’ as in LACE refers to the subgroup formed by stratification, whereas ‘local’ as in local average treatment effect refers to the subgroup of compliers (the individuals who would have the exposure present if they possess the genetic instrument, but would not otherwise). For a single instrument, the LACE estimate can be calculated using the ratio method: by dividing the genetic association with the outcome by the genetic association with the exposure [21].

We note that even though the instrument is not associated with confounders in the overall population, it may be associated in a subset of the population defined by stratification. The other two core IV assumptions (relevance and exclusion restriction) should hold in any subset of the population.

We denote the genetic instrument as *Z*, the exposure as *X*, the outcome as *Y*, and confounders of the exposure–outcome association (assumed unmeasured) as *U*. If there are multiple genetic variants that are valid instruments, these can be combined into a weighted score, which can then be used as the genetic instrument [22]. We first introduce the residual and doubly-ranked stratification methods, and then explore their properties in a simulation study and an applied analysis.

### Residual stratification method

Under the stratification assumption that the effect of the genetic instrument on the exposure is linear and homogeneous, the residual from regression of the exposure on the instrument will represent the value of the exposure as if the genetic instrument took the value zero. This variable, known as the ‘residual exposure’, is typically highly correlated with the exposure, as genetic instruments typically do not explain a large proportion of variance in the exposure. However, if we considered stratifying on the exposure directly, the distribution of the instrument would be different in the various strata. Values of the genetic instrument corresponding to increased levels of the exposure would be more common in strata with greater levels of the exposure. In contrast, not only is the residual exposure uncorrelated with the instrument, such that the instrument should be distributed on average similarly in the different strata; but, under the linearity and homogeneity assumptions, the functional dependence of the residual exposure on the instrument is broken. As the exposure is a common effect of the genetic instrument and confounders, the genetic instrument and confounders will be correlated within strata of the exposure; this is an example of collider bias [13]. However, as the residual exposure is not an effect of the genetic instrument, the genetic instrument and confounders will remain uncorrelated within strata of the residual exposure. We can therefore obtain LACE estimates within strata of the residual exposure. Individuals in each stratum would have similar values of the exposure if their instrument were set to the same fixed value.

### Doubly-ranked stratification method

**Fig 2:**
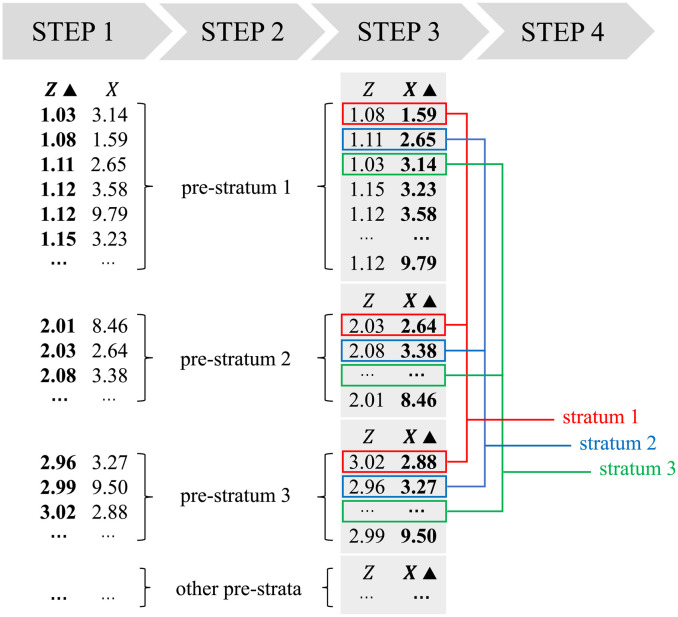
Schematic diagram illustrating the doubly-ranked stratification method. The stratification can be achieved in four steps accordingly. Step 1: sort the population according to the instrument *Z*; Step 2: build pre-strata according to the sorted *Z* values; Step 3: sort within each pre-stratum according to the exposure *X*; Step 4: select the first individuals from each pre-stratum into stratum 1, the second individuals in each pre-stratum into stratum 2, and so on.

A diagram outlining the doubly-ranked method is provided as Fig 2. For simplicity of explanation, we initially assume that we have a sample size of 1000, and the instrumental variable takes 100 different values 10 times each: that is, 10 individuals have *Z* = 1, 10 individuals have *Z* = 2, and so on. We first rank individuals into 100 pre-strata according to their level of the instrument, such that all those in pre-stratum *j* have *Z* = *j*. Then, within each pre-stratum we rank individuals according to their level of the exposure, and divide into strata. The first stratum consists of the individual who has the lowest value of the exposure in pre-stratum 1, the individual who has the lowest value of the exposure in pre-stratum 2, the individual who has the lowest value of the exposure in pre-stratum 3, and so on. The second stratum consists of the individual who has the second lowest value of the exposure in pre-stratum 1, the individual who has the second lowest value of the exposure in pre-stratum 2, the individual who has the second lowest value of the exposure in pre-stratum 3, and so on. We end up with 10 strata, each of which contains one individual from pre-stratum 1, one individual from pre-stratum 2, one individual from pre-stratum 3, and so on up to pre-stratum 100. The method is named doubly-ranked due to the two stratifications based on successive rankings: first, stratification into pre-strata based on the levels of the instrument; and then stratification into final strata based on the levels of the exposure within each pre-stratum.

In this simplified example, the distribution of the instrument is identical in each stratum by design; all strata have one individual with each value of the instrument from 1 to 100. As the stratification variable is not correlated with the instrument, the instrument should remain uncorrelated with confounders within each stratum. By construction, the average level of the exposure is increasing across the different strata. Hence, LACE estimates can be obtained within these strata which inform the investigator about average treatment effects (or local average treatment effects) for subsets of the population with different average levels of the exposure.

Suppose now that the instrument takes different values for a sample size of *N* = *J* × *K*, and we want to divide the population into *J* strata each containing *K* individuals. The doubly-ranked method works similarly as before: first, we rank individuals based on their level of the instrument, and divide into *K* pre-strata each containing *J* individuals; then we rank individuals within each pre-stratum based on their level of the exposure. We put the individuals with the lowest level of the exposure from each pre-stratum into stratum 1, the individuals with the second lowest level of the exposure from each pre-stratum into stratum 2, and so on. In this situation, the distribution of the instrument will not be exactly identical within each of the strata, but they should be similar by construction. Provided the effect of the instrument on the exposure is not too strong, correlation between the instrument and stratification will be low. Moreover, there is no functional dependence of stratification on the instrument. So, for a valid instrument, we expect correlation between the instrument and the confounders within the strata to be negligibly low. If some individuals have exactly the same value of the instrument or exposure, we break ties at random.

If the number of strata is small, or the instrument takes a small number of values, then rather than taking pre-strata of the same size as the number of strata, we can instead take pre-stratum size as a multiple of the number of strata. Say we want two strata, we can construct pre-strata of size 20, and then the 10 individuals with the lowest levels of the exposure from each pre-stratum are selected into stratum 1, and the 10 individuals with the highest levels of the exposure from each pre-stratum are selected into stratum 2. This should reduce the variability of stratum membership and ensure that the strata differ more strongly with respect to levels of the exposure, as with small pre-strata, we may find that all individuals in the pre-stratum have high (or low) levels of the exposure.

As in the residual method, we can obtain LACE estimates within strata of the population. As the method allows the genetic effect on the exposure to vary, we calculate the genetic associations with the exposure (and outcome) separately in each stratum to obtain the LACE estimates. For comparability of the methods, here we also estimate the genetic associations with the exposure within each stratum for the residual stratification method; if the effect of the genetic variant on the exposure is truly homogeneous, then it could be estimated more precisely in the whole population.

### Rank-preserving assumption

**Fig 3:**
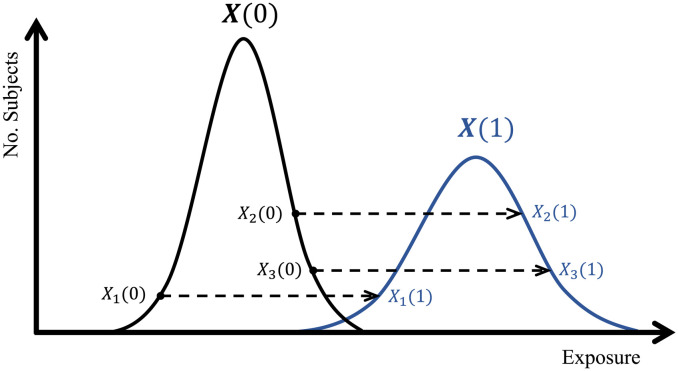
Diagram illustrating the rank preserving assumption for a dichotomous instrumental variable *Z* ∈ {0, 1} with counterfactual exposure distributions *X*(0) (the black group) and *X*(1) (the blue group). The dashed arrow represents the one-to-one mapping from the counterfactual exposure value with *Z* = 0 to the counterfactual covariate value with *Z* = 1.

The aim of the doubly-ranked method is to divide individuals into strata based on the ranking of their exposure value for their observed value of the instrument. The rank-preserving assumption states that an individual’s exposure ranking would be the same at all values of the instrument. To explain this assumption, we initially assume that the instrument is dichotomous, taking values 0 and 1, such that there are two counterfactual distributions of the exposure, one for each value of the instrument. We assume that an individual with instrument value *Z* = 0 at the 10th percentile of the counterfactual distribution of the exposure for *Z* = 0 would be at the 10th percentile of the counterfactual distribution of the exposure for *Z* = 1 if they had instead an instrument value *Z* = 1; and similarly for any other percentile of the exposure distribution. This assumption is illustrated in Fig 3.

By first stratifying individuals into pre-strata based on their value of the instrument, and then into strata based on their value of the exposure within each pre-stratum, we construct strata with similar exposure ranking values. As the exposure ranking values are independent of the instrument, if the instrumental variable assumptions are satisfied in the population as a whole, then they will be satisfied in subgroups stratified on the exposure ranking. If the instrument is not dichotomous, but continuous, then there are counterfactual distributions of the exposure at each value of the instrument; the rank-preserving assumption is that each individual’s exposure value would be at the same percentile of the relevant counterfactual distribution no matter their value of the instrument. The doubly-ranked method will work well if the pre-strata have the same or similar values of the instrument, as in this case, the ordering of individuals within each pre-stratum will correspond to their counterfactual exposure rankings. The doubly-ranked method will work less well if instrument values vary strongly within pre-strata, and the effect of the instrument on the exposure is strong. The linear and homogeneous assumption made by the residual stratification method is a special case of the rank-preserving assumption, and so the rank-preserving assumption is strictly weaker than the linear and homogeneous assumption.

### Coarsened exposure values

The residual stratification method requires values of the exposure to be known precisely, otherwise it is not possible to calculate the residual exposure in a way that is not causally dependent on the instrument values. However, in practice many exposure variables are coarsened [23]. For example, alcohol intake is often measured in categories (such as 0–5 g/day, 5–10 g/day, and so on), not absolute amounts; age at menarche is typically reported as a whole number of years; and manual blood pressure measurements are often preferentially reported as multiples of 5. This is a particular problem when the categorization is more coarse than the effect of the instruments; for example, sleep duration is often reported as a whole number of hours, but the average effect of genetic variants on sleep duration is far less than 1 hour. This means that boundaries between strata would either divide individuals according to their exposure values, introducing collider bias, or else they would divide individuals for a given value of the exposure according to their instrument values, creating strata with irregular distributions of the instrument.

In contrast, the doubly-ranked method should be able to form meaningful strata even if the exposure is coarsened. A further practical feature of the doubly-ranked method is that strata are equal in size even if the distribution of the exposure is irregular.

If the exposure is coarsened to take a small number of values, then it is not possible to divide the population into a large number of strata in a meaningful way. In an extreme case, it may be that all the individuals in a stratum have the same value of the exposure, in which case the relevance assumption does not hold. There is also a possible violation of the exchangeability assumption when coarsening of the exposure leads to patterns arising within the strata. We have developed a Gelman–Rubin uniformity statistic to assess whether clumps in the coarsened exposure distribution (that is, groups of individuals with the same coarsened exposure value) are distributed uniformly within the strata; high values of this statistic indicate potential violation of the exchangeability assumption, and suggest the use of fewer strata (see Text A in S1 Text).

A related situation is when the data are not coarsened uniformly, but extreme values of the exposure are trimmed or winsorized. In this case, estimates for the middle strata (in which the data are not altered) are unaffected, and estimates for the exposure associations in boundary strata will be underestimated, with the extent of underestimation depending on the degree of trimming. However, unless trimming is performed on a substantial fraction of the data, it should not affect assignment into strata using the doubly-ranked method, as it does not change the ranking of most exposure measurements. A further situation is when the exposure has a natural maximum or minimum value, such as a minimum of zero for many biomarkers. In this case, there is no reason why the doubly-ranked method would work differently provided that the rank-preserving assumption is satisfied. However, the linear and homogeneous assumption cannot be satisfied in such a situation, as discussed in the context of the applied example.

## Verification and comparison

### Simulation study

We explore how the residual and doubly-ranked stratification methods perform in a range of simulation scenarios. We consider three causal relationships between the exposure and the outcome:

1. No causal effect of the exposure on the outcome: *Y* = *U* + *ϵ*_*Y*_.
2. U-shaped causal effect of the exposure on the outcome: *Y* = 0.1*X*^2^ + *U* + *ϵ*_*Y*_.
3. Threshold causal effect of the exposure on the outcome: $Y=\{\begin{array}{cc} U+\epsilon_{Y} & \;\text{for}\;X\leq 0\\ -0.1X^{2}+U+\epsilon_{Y} & \;\text{for}\;X>0 \end{array}$

In the first case, the causal effect of the exposure on the outcome is zero throughout. In the second case, the causal effect is negative for negative exposure values and positive for positive exposure values. In the third case, the causal effect is zero for negative exposure values and negative for positive exposure values.

We also consider four models for the effect of the instrument on the exposure:

A. Linearity and homogeneity: *X* = 0.5*Z* + *U* + *ϵ*_*X*_.
B. Non-linearity and homogeneity: $X=\{\begin{array}{cc} 0.5Z+2Z^{3}+2+U+\epsilon_{X} & \;\text{for}\;Z\leq -1\\ 0.5Z+U+\epsilon_{X} & \;\text{for}\;Z>-1 \end{array}$.
C. Linearity and heterogeneity: *X* = −10 + (1.5 + 0.4*U*)(*Z* + 5) + *U* + *ϵ*_*X*_
D. As scenario A, but the exposure is coarsened by being rounded to the nearest integer value.

In model A, the effect of the instrument is linear and homogeneous. In model B, it is non-linear as there is a power change at *Z* = −1. In model C, it is heterogeneous, with the effect depending on the value of the confounder *U*. In model D, the effect is linear and homogeneous for the original values of the exposure, but non-homogeneous for the coarsened values.

We consider 12 scenarios, comprising all combinations of exposure–outcome relationships and instrument–exposure models. We simulate data on 10 000 individuals in each dataset, and consider 1000 simulated datasets per scenario. In each scenario, we simulate variables from independent normal distributions: $Z∼N(0,0.5^{2})$, $U∼N(0,1^{2})$, $\epsilon_{X}∼N\left(0,1^{2}\right)$, and $\epsilon_{Y}∼N\left(0,1^{2}\right)$, where *ϵ*_*X*_ and *ϵ*_*Y*_ are independent error terms for the exposure *X* and outcome *Y* respectively. The average proportion of variation in the exposure explained by the instrument in each scenario is roughly 5%. We divide each dataset into 10 strata of 1000 individuals using both of the stratification methods, and calculate LACE estimates in each stratum. We also assess genetic associations with the exposure in each stratum to see whether these follow the expected pattern.

To assess the performance of these methods with different types of instrument, we repeated these simulation scenarios with a single dichotomous instrument, and with a small number of independent instruments representing different genetic variants. We also repeated the simulation study under model A using a continuous instrument with three different confounder–outcome relationships, to assess the performance of the methods with different confounding structures. Details of these scenarios are given in Text B in S1 Text.

### Comparison with other methods

Various methods have been proposed for non-linear instrumental variable analysis [24, 25]. Many such methods perform a two-stage procedure, in which the first stage fits a model relating the exposure to the instrument, and the second stage fits a model relating fitted values of the exposure to the outcome in a flexible modelling framework [26]. However, in Mendelian randomization, the instrument often takes a small number of discrete values, meaning that the fitted values of the exposure only take a small number of values. Additionally, many genetic instruments explain only a small proportion of variance in the exposure, and so fitted values of the exposure are only available for a narrow range of the exposure distribution. Estimating causal effects on the outcome for a relevant range of the exposure distribution would require extrapolation in the second-stage model. A recently proposed method in this category is the Deep Learning for IV Regression (DeLIVR) method [27], designed for transcriptome-wide association study (TWAS) data. However, while this method may work reasonably in the context of TWAS, where genetic variants often explain a substantial portion of variance in the exposure, it is unlikely to work well in Mendelian randomization contexts such as the alcohol example in this study, where the genetic variants explain around 0.7% of the variance in the exposure. A related method to the residual stratification method is PolyMR. This method also calculates residual values of the exposure, similar to the residual stratification method, but it uses these residual values to fit a parametric model for the outcome [28]. It is an example of a control function approach [29]. As it uses residual values of the exposure, we expect the PolyMR method to be sensitive to violations of the linear and homogeneity assumptions for the instrument effect on the exposure.

As the PolyMR method does not provide stratum-specific estimates, we cannot compare its performance to the stratification methods for estimating stratum-specific effects. Instead, we consider an aspect of the problem that is assessed by both PolyMR and the stratification methods: whether a linear or non-linear model is preferred by the method. We assessed linearity in the stratified methods by calculating 10 stratum-specific estimates and calculating a heterogeneity statistic similar to Cochran’s Q statistic, and comparing to a *χ*^2^ distribution on 9 degrees of freedom [30]. We assessed linearity in the PolyMR method by a likelihood ratio test comparing the best-fitting model from the PolyMR method against a linear model. We simulated 1000 datasets in a range of scenarios, and report the proportion of datasets in which the null hypothesis of a linear model was rejected. Further details of these methods are provided in Text B in S1 Text.

### Results

#### Simulation study

**Fig 4:**
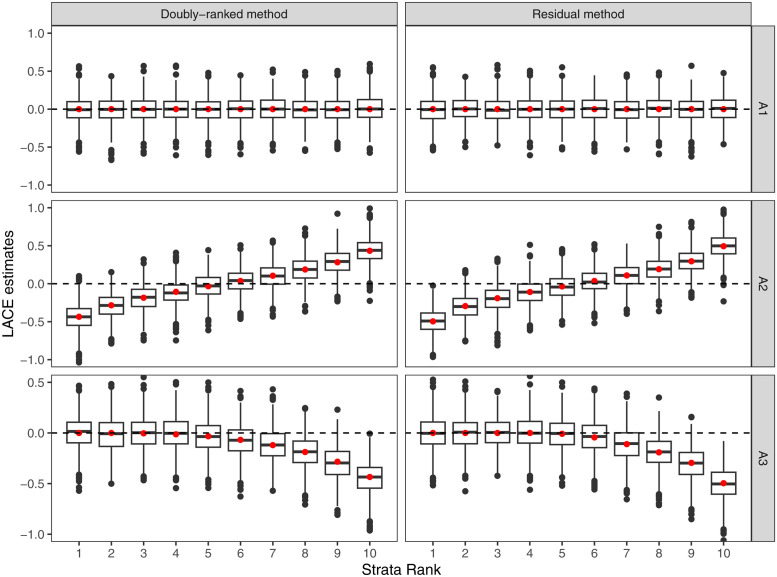
Results of the doubly-ranked method and residual method for model A (linearity and homogeneity) with three different causal relationship between the exposure and the outcome (denoted by A1, A2, A3). Boxplot results represent the LACE estimates within the 10 strata. Red points represent the target causal effects within strata. Box indicates lower quartile, median, and upper quartile; error bars represent the minimal and maximal data point falling in the 1.5 interquartile range distance from the lower/upper quartile; estimates outside this range are plotted separately.

**Fig 5:**
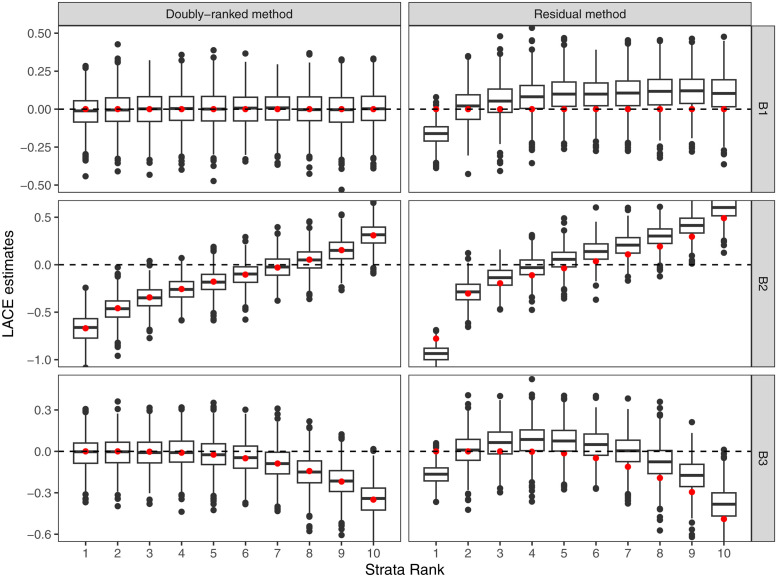
Results of the doubly-ranked method and residual method for model B (nonlinearity and homogeneity) with three different causal relationship between the exposure and the outcome (denoted by B1, B2, B3). Boxplot results represent the LACE estimates within the 10 strata. Red points represent the target causal effects within strata. Box indicates lower quartile, median, and upper quartile; error bars represent the minimal and maximal data point falling in the 1.5 interquartile range distance from the lower/upper quartile; estimates outside this range are plotted separately.

**Fig 6:**
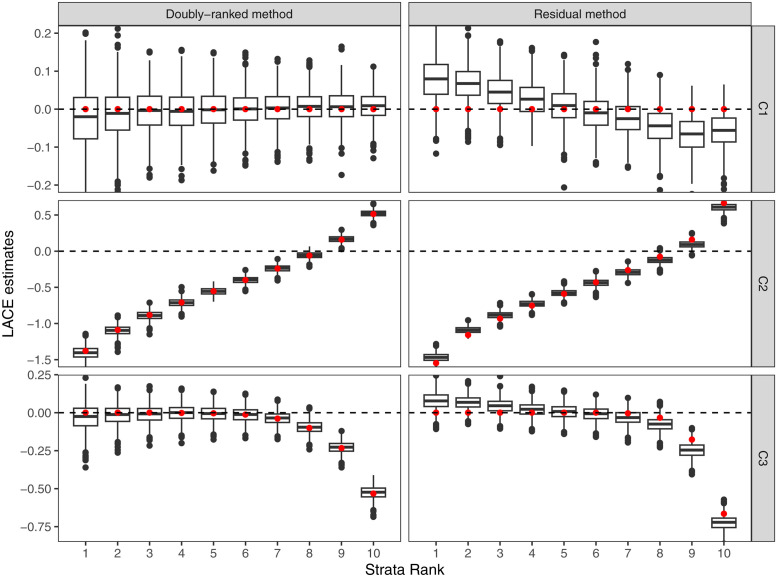
Results of the doubly-ranked method and residual method for model C (linearity and heterogeneity) with three different causal relationship between the exposure and the outcome (denoted by C1, C2, C3). Boxplot results represent the LACE estimates within the 10 strata. Red points represent the target causal effects within strata. Box indicates lower quartile, median, and upper quartile; error bars represent the minimal and maximal data point falling in the 1.5 interquartile range distance from the lower/upper quartile; estimates outside this range are plotted separately.

**Fig 7:**
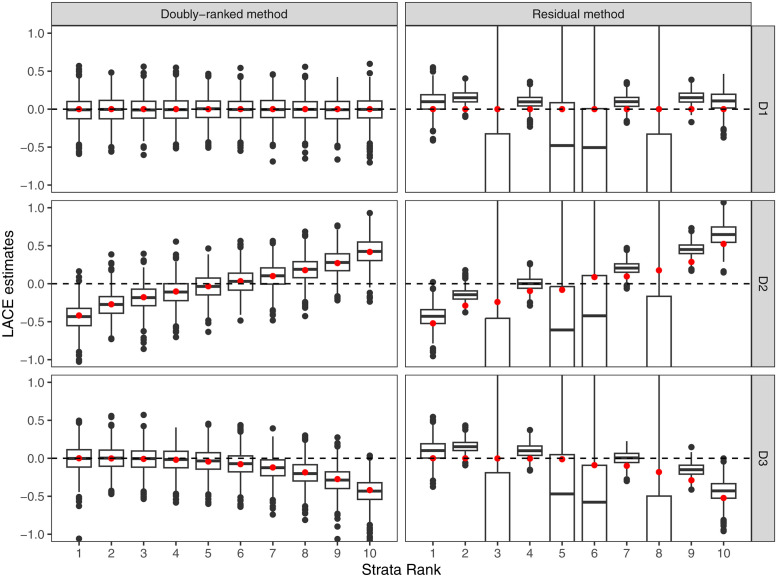
Results of the doubly-ranked method and residual method for model D (coarsened exposures) with three different causal relationship between the exposure and the outcome (denoted by D1, D2, D3). Boxplot results represent the LACE estimates within the 10 strata. Red points represent the target causal effects within strata. Box indicates lower quartile, median, and upper quartile; error bars represent the minimal and maximal data point falling in the 1.5 interquartile range distance from the lower/upper quartile; estimates outside this range are plotted separately.

Results from the residual stratification and the doubly-ranked method are displayed in Figs 4–7. For each scenario, we provide a boxplot of LACE estimates in each stratum. Median estimates from the residual stratification method are close to the average true effect values for that stratum in Scenarios A1, A2, and A3, where the effect of the instrument on the exposure is linear and homogeneous. However, in other scenarios, there is notable bias and distortion of the shape of the exposure-outcome causal relationship, even in Scenarios B1 and C1, where the true causal effect is null, and in Scenario D1, where the effect of the instrument on the exposure is linear and homogeneous, but the exposure is rounded to the nearest integer. The imprecise estimates in Scenario D1 correspond to strata where the majority of people have similar estimates for the exposure, and so the genetic association with the exposure in that stratum is weak. In contrast, median estimates from the doubly-ranked stratification method are close to the true values throughout.

Table 1 provides two further summaries of the simulation results: the mean squared error (MSE) of estimates summed across the 10 strata, and the coverage of estimates, representing the proportion of LACE estimates for which the 95% confidence interval included the average causal effect for that stratum, defined as the weighted integral of the derivative of the function relating the exposure to the outcome with the weight function estimated by the instrument and exposure values of individuals in each stratum (as shown and derived in Text C in S1 Text). We refer to this average causal effect as the target causal effect. When the effect of the instrument on the exposure is linear and homogeneous (model A), both methods perform similarly. For other models, the doubly-ranked method has better performance than the residual method in terms of both MSE and coverage. For models B and C, the residual method has inflated type I error rates while the doubly-ranked method has appropriate type I error rates. In addition, the doubly-ranked method has prominently better performance in the coarsened exposure case (model D), whereas residual methods do not work well even under the linearity and homogeneity model. We note that the Gelman–Rubin uniformity statistic was below the threshold value of 1.02 in all strata for the coarsened exposure.

**Table 1:** Summary of simulation study results: Mean squared errors (MSE) and coverage of the 95% confidence interval for the residual and doubly-ranked stratification method in each scenario. MSE is calculated as an average across estimates in all 10 strata, and the results are averaged across 1000 datasets per scenario.

Scenario	Residual method		Doubly-ranked method	
	MSE	Coverage	MSE	Coverage
A1	0.026	0.947	0.027	0.948
A2	0.025	0.948	0.026	0.951
A3	0.026	0.945	0.025	0.951
B1	0.023	0.807	0.013	0.951
B2	0.023	0.832	0.014	0.958
B3	0.024	0.807	0.013	0.946
C1	0.005	0.810	0.003	0.947
C2	0.005	0.865	0.003	0.975
C3	0.005	0.816	0.003	0.946
D1	2.603	0.801	0.028	0.947
D2	4.533	0.794	0.027	0.951
D3	2.458	0.799	0.027	0.947

Additionally, we assessed the genetic associations with the exposure within strata defined by the two methods. Results are shown in Figs A-D in S1 Text. Genetic associations with the exposure in strata defined by the doubly-ranked method follow the expected pattern: homogeneous for models A, B, and D, but monotone increasing for model C. In contrast, genetic associations with the exposure in strata defined by the residual method are similar for strata 2 to 9 in all non-coarsened exposure scenarios, even for model C, where they should not be similar. With a coarsened exposure (model D), genetic associations with the exposure in strata defined by the residual method are highly irregular, providing further evidence on the unreliability of this method in the coarsened exposure scenario. Our results suggest the approach of assessing stratum-specific genetic associations with the exposure is only reliable as a test of homogeneity of the instrument effect using the doubly-ranked method.

To assess the performance of the method with different types of instrument, we performed additional simulation scenarios with a single dichotomous instrument, and with a small number of independent instruments representing different genetic variants. Results are shown in Figs E-H in S1 Text for the dichotomous instrument, and in Figs I-L in S1 Text for the independent instruments. We see that the doubly-ranked method performed similarly well in these scenarios. To assess the performance of the method with different confounder–outcome models, we repeated the simulation under model A for three different confounder–outcome models. Results are shown in Fig M and Table A in S1 Text. Again, the doubly-ranked method performed similarly well in these scenarios.

#### Comparison with other methods

Results comparing the PolyMR method to the stratification methods are provided in Table 2. In all scenarios in which the exposure has a null causal effect on the outcome, the proportion of datasets in which a non-linear model was preferred over a linear model was close to the nominal 5% level for the doubly-ranked method. However, for the residual and PolyMR methods, this proportion was close to 5% in Scenario A1 for both methods, and Scenario D1 for the PolyMR method. In other scenarios, a non-linear effect was reported even though the true effect was null. In all scenarios in which the exposure has a quadratic effect on the outcome, the proportion of datasets in which a non-linear model was preferred over a linear model was clearly above 5% for all methods. While the residual and PolyMR methods generally had greater empirical power than the doubly-ranked method, this comparison is largely unfair as the residual and PolyMR methods did not maintain nominal coverage levels under the null.

**Table 2:** Comparison of the stratification and PolyMR methods: Proportion of datasets in which the null hypothesis of homogeneity of stratum-specific estimates (for stratification methods) or linearity (for PolyMR) was rejected at a 5% significance level in various scenarios with a null causal effect (scenarios A1, B1, C1, D1, and A1+U3; proportion represents empirical Type I error rate) and with a quadratic causal effect (scenarios A2, B2, C2, D2, and A2+U3; proportion represents empirical power). Note that scenarios A1+U3 and A2+U3 are equivalent to scenarios A1 and A2, except that the confounder effect on the outcome is non-linear (see Text B in S1 Text for details).

Scenario	Dichotomous instrument			Continuous instrument		
	Doubly-ranked	Residual	PolyMR	Doubly-ranked	Residual	PolyMR
A1	0.045	0.056	0.047	0.053	0.062	0.044
B1	0.045	0.056	0.047	0.047	0.661	0.990
C1	0.041	0.468	0.868	0.050	0.629	1.000
D1	0.047	0.531	0.051	0.066	0.165	0.050
A1+U3	0.048	0.117	0.693	0.047	0.113	0.733
A2	0.850	0.952	0.976	0.929	0.988	0.994
B2	0.850	0.952	0.976	0.999	1.000	1.000
C2	1.000	1.000	1.000	0.999	1.000	1.000
D2	0.861	1.000	0.973	0.932	1.000	0.995
A2+U3	0.212	0.447	0.877	0.233	0.509	0.916

## Applications

We implement the residual and doubly-ranked stratification methods to study the causal effect of alcohol intake on SBP. Previous Mendelian randomization analyses have suggested that alcohol intake has positive causal effects on hypertension risk and systolic blood pressure [31–33], although the shape of the causal relationship has not been considered using this approach. We take data from UK Biobank, a prospective cohort study of around half a million UK residents aged 40 to 69 years at baseline, recruited in 2006–2010 from across the United Kingdom [34]. We consider 385 000 unrelated individuals of European ancestries, who passed various quality control filters as described previously [35]. A small number of individuals (2067) were dropped from the analysis at random to obtain equally-sized strata, facilitating comparison of stratum-specific estimates for this illustrative example.

We first construct a weighted genetic score for each individual from 93 genetic variants which have previously been shown to be associated with alcohol intake in 941 280 individuals at a genome-wide level of statistical significance [36]. Only around 124 590 individuals in this analysis were UK Biobank participants, minimizing bias due to sample overlap [37]. The weighted genetic score was centred to have mean zero, so that the mean of the residual exposure was the same as the mean of the exposure. Alcohol consumption was calculated for each participant based on self-reported data on consumption frequency for various alcoholic drinks as described previously [38], and is measured in units of g/day. We obtain LACE estimates using each stratification method for 77 strata, each of which contains 5000 individuals. These are plotted against the average value of the exposure in each stratum, thus providing insight into the shape of the causal relationship. We also provide LACE estimates for tenths of the population.

### Results

Even though the level of alcohol intake is a continuous measure, it has a natural left truncation at zero g/day. Additionally, while detailed information on various categories of alcohol consumption is available in UK Biobank, the questionnaire nature of the data means that several individuals have the same reported value for alcohol intake. Overall, 5284 unique values of alcohol intake were represented in the data, and 21 340 individuals reported zero alcohol intake. This therefore represents a coarsened exposure scenario. LACE estimates represent change in SBP in mmHg units per 1 g/day increase in genetically-predicted values of alcohol intake.

**Fig 8:**
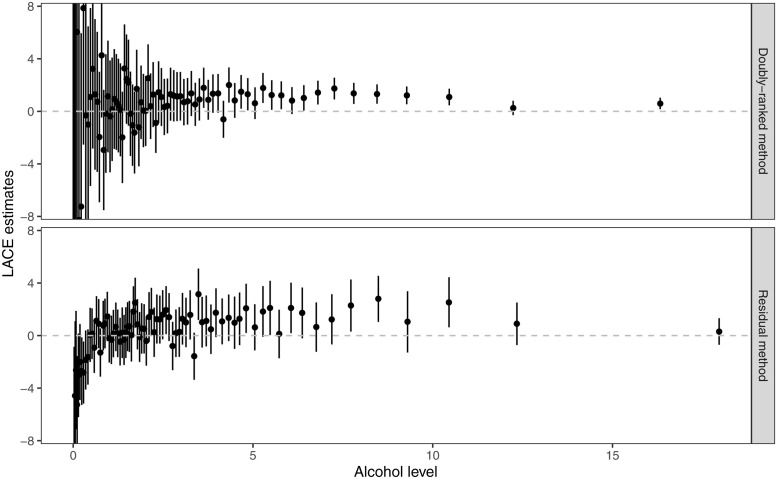
LACE estimates of alcohol intake on SBP from the two stratification methods (residual method and doubly-ranked method) against average levels of alcohol intake in the 77 strata. The error bars represent the 95% confidence interval for each stratum-specific estimate.

Graphs of LACE estimates from the two stratification methods plotted against average alcohol intake in that stratum are presented in Fig 8. For the residual stratification method, most estimates in the low alcohol range are in the negative direction. But residual alcohol is negative for some individuals with zero alcohol consumption; this value does not have a natural interpretation, and we cannot conceive of individuals having a negative alcohol consumption. It is logically impossible that the genetic effect on alcohol consumption is homogeneous in the population, and so the constant genetic effect assumption is violated. The effect of the genetic variants for zero consumption individuals cannot be positive, as this would imply negative consumption if they had a different value of the genetic variants. Therefore, estimates in low consumption strata from the residual method are unreliable.

In contrast, LACE estimates from the doubly-ranked method are generally positive throughout. For moderate to heavy drinkers (>5 g/day alcohol intake), estimates from the two methods are similar, but those from the doubly-ranked method have less uncertainty and a greater proportion of 95% confidence intervals that exclude the null. This may be due to the increasing genetic associations with the exposure within strata for the doubly-ranked method (Fig N in S1 Text), leading to more precise ratio estimates as observed for model C of the simulation study.

**Fig 9:**
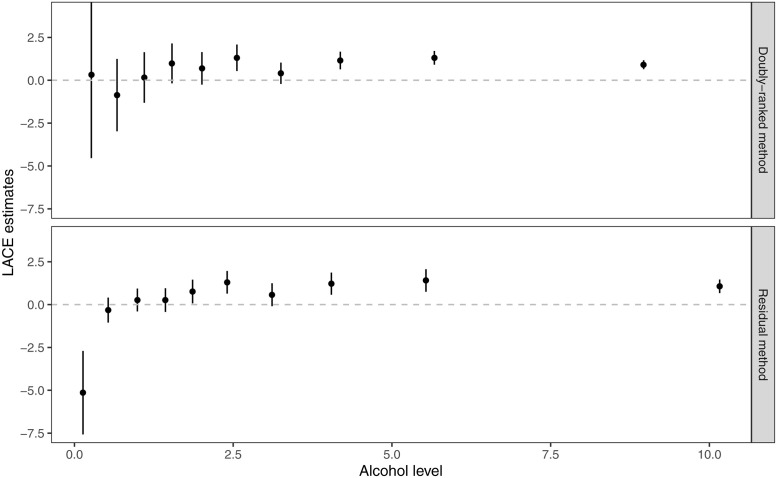
LACE estimates of alcohol intake on SBP from the two stratification methods (residual method and doubly-ranked method) against average levels of alcohol intake in the 10 strata. The error bars represent the 95% confidence interval for each stratum-specific estimate.

A similar pattern of LACE estimates is seen when dividing the population into tenths using both methods: the LACE estimate is -5.14 (95% confidence interval -7.57 to -2.70) in the lowest decile group for the residual method, and 0.32 (95% confidence interval -4.54 to 5.18) for the doubly ranked method (Table 3 and Fig 9). The negative estimate in the lowest quantile for the residual method is biologically implausible. It is also implausible that the causal estimate is stronger in the lowest decile group than in the other groups, as the genetic change in alcohol consumption at low consumption levels is much smaller than at high consumption levels, and small increases in alcohol consumption at low consumption levels would be quickly cleared by the body, and so would be expected to have a lesser effect. In contrast, the estimates from the doubly-stratified method are close to the null in the lowest quantiles, and suggest a positive effect from the fourth quantile onwards, with significant positive estimates in strata 6, 8, 9, and 10 (*p* < 0.05). This is biologically plausible, as alcohol will remain in the system longer for moderate to heavy drinkers, and so the effect of a 1 g/day increase will likely be greater than for light drinkers [39]. Moderate to heavy drinkers are also more likely to include episodic drinkers (so called ‘binge drinkers’), for whom alcohol potentially has a more strongly harmful effect [40].

**Table 3:** Summary of applied investigation results: The mean alcohol intake (g/day) with its interquartile interval, LACE estimate and 95% confidence interval (mmHg change in systolic blood pressure per 1 g/day increase in genetically-predicted alcohol intake) in each stratum using the residual method and doubly-ranked method. IQR represents the interval for the interquartile range in each stratum. CI represents the 95% confidence interval.

Stratum	Residual method			Doubly-ranked method		
	Mean (IQR)	Estimate	95% CI	Mean (IQR)	Estimate	95% CI
1	0.13(0.00,0.24)	-5.14	(-7.57,-2.70)	0.27(0.00,0.45)	0.32	(-4.54,5.18)
2	0.53(0.33,0.77)	-0.32	(-1.05,0.41)	0.67(0.24,1.03)	-0.87	(-2.98,1.25)
3	0.99(0.77,1.21)	0.27	(-0.39,0.94)	1.10(0.65,1.51)	0.16	(-1.31,1.64)
4	1.43(1.29,1.56)	0.26	(-0.43,0.95)	1.54(1.03,1.93)	0.98	(-0.18,2.15)
5	1.86(1.69,2.06)	0.76	(0.07,1.45)	2.01(1.47,2.46)	0.70	(-0.25,1.64)
6	2.40(2.21,2.60)	1.30	(0.64,1.96)	2.56(1.83,3.09)	1.31	(0.54,2.08)
7	3.11(2.89,3.35)	0.58	(-0.09,1.24)	3.25(2.31,3.94)	0.41	(-0.21,1.03)
8	4.05(3.71,4.40)	1.22	(0.58,1.87)	4.19(3.09,5.09)	1.15	(0.64,1.67)
9	5.54(5.09,6.04)	1.41	(0.75,2.07)	5.67(4.00,6.86)	1.31	(0.91,1.71)
10	10.16(7.60,11.41)	1.07	(0.67,1.46)	8.96(5.86,10.91)	0.91	(0.65,1.17)

Genetic associations with the exposure in strata are displayed in Fig N in S1 Text. While genetic associations in strata defined by the residual method were similar for strata 2 to 9, we showed in the simulation study that this is not a reliable way of assessing variability in instrument strength. In contrast, genetic associations with the exposure in strata defined by the doubly-ranked method were monotone increasing across the strata, similar to model C in the simulation study. This suggests the genetic variants have a stronger effect on alcohol intake for higher levels of alcohol consumption. This is likely due, at least in part, to alcohol consumption having a natural minimum value of zero, as well as potentially due to the genetic variants having a proportional effect on increasing alcohol consumption. This provides further empirical evidence that the doubly-ranked method is more appropriate for this example. Values of the Gelman–Rubin uniformity statistic were below 1.02 for both the stratification into 77 strata and into 10 strata, suggesting that the number of strata is not excessive given the degree of coarsening. The distribution of the exposure in strata defined by the doubly-ranked method is normally broader than that by the residual method.

A potential limitation of the analysis is that the zero alcohol consumption group contains both never-drinkers and ex-drinkers. However, in contrast to chronic disease outcomes, the impact of alcohol consumption on SBP is likely to be short-term in nature [41]. Therefore any lifelong effect of exposure to alcohol in ex-drinkers is less likely to impact findings for this outcome.

## Discussion

Stratified non-linear Mendelian randomization is an extension to standard Mendelian randomization that stratifies the population into subgroups with different average levels of the exposure, and then performs separate instrumental variable analyses in each stratum of the population. In this paper, we have proposed the doubly-ranked method, a non-parametric method for constructing strata of the population, such that the strata are uncorrelated with the instrument, and the average level of the exposure is increasing across strata. In the case where the instrument takes a fixed value in each pre-stratum, the correlation between the instrument and stratification is exactly zero. In other cases, this correlation should be close to zero. In either case, stratification using this method should not induce substantial collider bias, and so if the genetic instrument is valid in the population as a whole, it should be valid in strata of the population defined using the doubly-ranked method.

We validated the doubly-ranked method in a simulation study for a range of scenarios, including various models for the effect of the exposure on the outcome (null, U-shaped, threshold) and for the effect of the instrument on the exposure, different instrument types, and different models for the effect of the confounder on the outcome. The previously proposed residual stratification method provided unbiased estimates when the effect of the instrument on the exposure was linear and homogeneous, but otherwise provided biased estimates with inflated Type I error rates. In contrast, the doubly-ranked stratification method provided unbiased estimates and appropriate coverage rates when the effect of the instrument was non-linear or heterogeneous. It also provided unbiased estimates when the exposure was coarsened by rounding its value to the nearest integer; another scenario that can lead to large bias for the residual method. The coarsened exposure scenario is particularly important in applied practice, as many exposures are reported as rounded values or in categories. We also assessed the performance of the doubly-ranked method in an applied example, which demonstrated evidence for an effect of alcohol on systolic blood pressure that was positive across strata at moderate to high levels of alcohol consumption, while the residual method suggested a negative effect at low levels of alcohol consumption that is not biologically plausible.

Aside from relaxing the parametric assumptions required by the residual method, the doubly-ranked method is able to deal with a wider set of exposures. A further feature is that strata formed by the doubly-ranked method are equally-sized, meaning that the precision of LACE estimates should be similar across the distribution of the exposure. A disadvantage of the method is that it is harder to stratify the population according to clinically-defined threshold values of the exposure, such as the World Health Organization categories for BMI (BMI < 18.5 kg/m^2^ is underweight, and so on). Similarly, it is difficult to define which individuals are selected into each stratum, and so whom each stratum-specific estimate relates to.

In addition to being used to estimate the non-linear relationship, the doubly-ranked method can also assess the homogeneity assumption. Under homogeneity, the genetic associations with the exposure in strata obtained from the doubly-ranked method should be consistent to a fixed value. Any inconsistent pattern, like the increasing pattern for the alcohol example, indicates the effect of the instrument on the exposure is heterogenous, and hence the doubly-ranked method should be used in preference to the residual method.

A limitation of the doubly-ranked method is that the division into strata can be extremely sensitive to the specification of the analytic dataset. The omission or inclusion of a small number of individuals in the dataset can result in large variability in the stratum-specific estimates, as different individuals are selected into the strata. While estimates remain unbiased, this variability is not desirable. A suggestion to reduce this variability is to consider random samples of the data, omitting a small number of individuals from the dataset (say, 10 individuals) at random in each iteration. Stratum-specific estimates can then be combined using Rubin’s rules.

Previous work has considered smoothing LACE estimates from the residual method using either a fractional polynomial or piecewise linear method [4]. These methods could equally be applied to LACE estimates from the doubly-ranked method. The result of these methods is a plot of the outcome against the exposure, which can more easily be compared to the shape of the association estimated in a conventional observational epidemiological analysis. The LACE estimates represent the gradient of this curve, as they represent the average causal effect at that level of the exposure, and so the plot of the LACE estimates against the exposure is the derivative of the plot of the outcome against the exposure. A plot of the LACE estimates against the exposure is less conventional, but it more clearly represents the quantities estimated in a non-linear Mendelian randomization investigation, which are the stratum-specific causal effects.

A perennial question is the choice of how many strata to consider in a given application. Aside from the recommendation not to include too many strata if the exposure is highly coarsened, consideration of the optimal number of strata depends on the shape of the causal relationship expected by the investigator, and the objective of the investigation. For example, if the investigator believes that the causal effect varies strongly at the top and/or bottom ends of the exposure distribution, then they should consider a large number of strata so that the lowest and/or highest strata mostly contain individuals at these exposure values. Otherwise, if the investigator wants more, less precise estimates, then they should include more strata; if they want fewer, more precise estimates, then they should include less strata.

In summary, this paper presents the doubly-ranked stratification method, a method for relaxing the parametric assumptions made in non-linear Mendelian randomization investigations. We recommend analysts consider using this method in preference to the residual stratification method, or at least as a sensitivity analysis to assess the dependence of findings on the linearity and homogeneity assumptions for the effect of the instrument on the exposure.

## Supporting information

S1 Text Supplementary materials. (PDF)
