# Supplementary material for: Relaxing parametric assumptions for non-linear Mendelian randomization using a doubly-ranked stratification method
Source: PLoS Genet. 2023 Jun 30;19(6):e1010823. doi: 10.1371/journal.pgen.1010823 (PMC10343089; doi:10.1371/journal.pgen.1010823)
Supplement: S1 Text — (PDF) [file pgen.1010823.s001.pdf]

Relaxing parametric assumptions for non-linear Mendelian randomization using a doubly-ranked stratification method SUPPORTING MATERIALS

Haodong Tian [1*] Amy M. Mason [2] Cunhao Liu [1]
Stephen Burgess [1,2]

[1] MRC Biostatistics Unit, University of Cambridge, Cambridge, UK

[2] British Heart Foundation Cardiovascular Epidemiology Unit,
Department of Public Health and Primary Care,
University of Cambridge, Cambridge, UK

June 13, 2023

The supplementary materials include the following supporting information:
Table A. Results for different confounder–outcome relationships.
Figs A-D. Genetic associations with the exposure for each scenario.
Figs E-H. Results from the stratification method with dichotomous instrument.
Figs I-L. Results from the stratification method with independent instruments.
Fig M. Results for different confounder–outcome relationships.
Fig N. Genetic associations with the exposure for the real example.
Text A. Exchangeability assessment for the coarsened exposure in each stratum.
Text B. Additional simulation scenarios.
Text C. Mathematical details for the causal effect in each stratum.

*Corresponding author. MRC Biostatistics Unit, University of Cambridge, East Forvie Building, Forvie Site, Robinson Way, Cambridge, CB2 0SR, UK. Email: haodong.tian@mrc-bsu.cam.ac.uk

Supplementary Table

	U1		U2		U3	
Causal Model	MSE	Coverage	MSE	Coverage	MSE	Coverage
1	0.047	0.951	0.021	0.951	0.095	0.951
2	0.046	0.957	0.021	0.959	0.093	0.961
3	0.046	0.948	0.022	0.946	0.098	0.947

Table A: Summary of simulation study results for different causal relationships (denoted by 1, 2, 3) and confounder–outcome relationships (denoted by U1, U2, U3): mean squared errors (MSE) and coverage of the 95% confidence interval for the residual and doubly-ranked stratification method in each scenario. Results are under the instrument–exposure model A with continous instruments. MSE is calculated as an average across estimates in all 10 strata, and the results are averaged across 1000 datasets per scenario.

Supplementary Figures

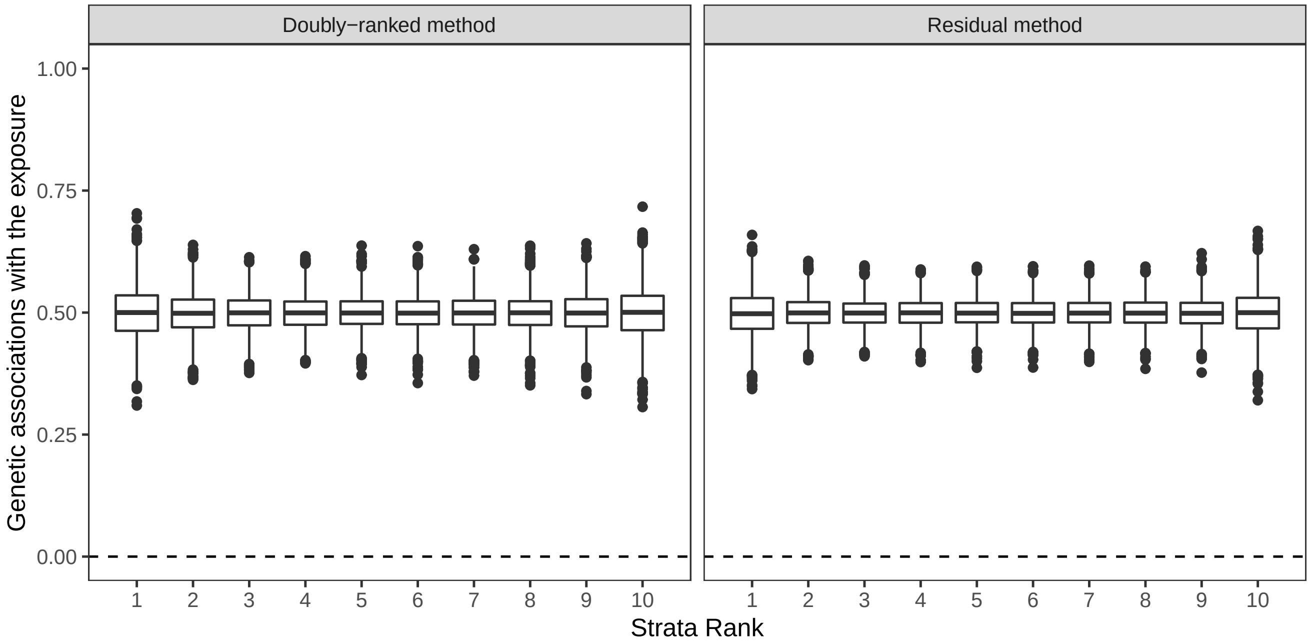

Fig A: Results of the doubly-ranked method and residual method for model A (linearity and homogeneity). Boxplot results represent the estimates of genetic associations with the exposure within the 10 strata under 3000 simulations. Box indicates lower quartile, median, and upper quartile; error bars represent the minimal and maximal data point falling in the 1.5 interquartile range distance from the lower/upper quartile; estimates outside this range are plotted separately.

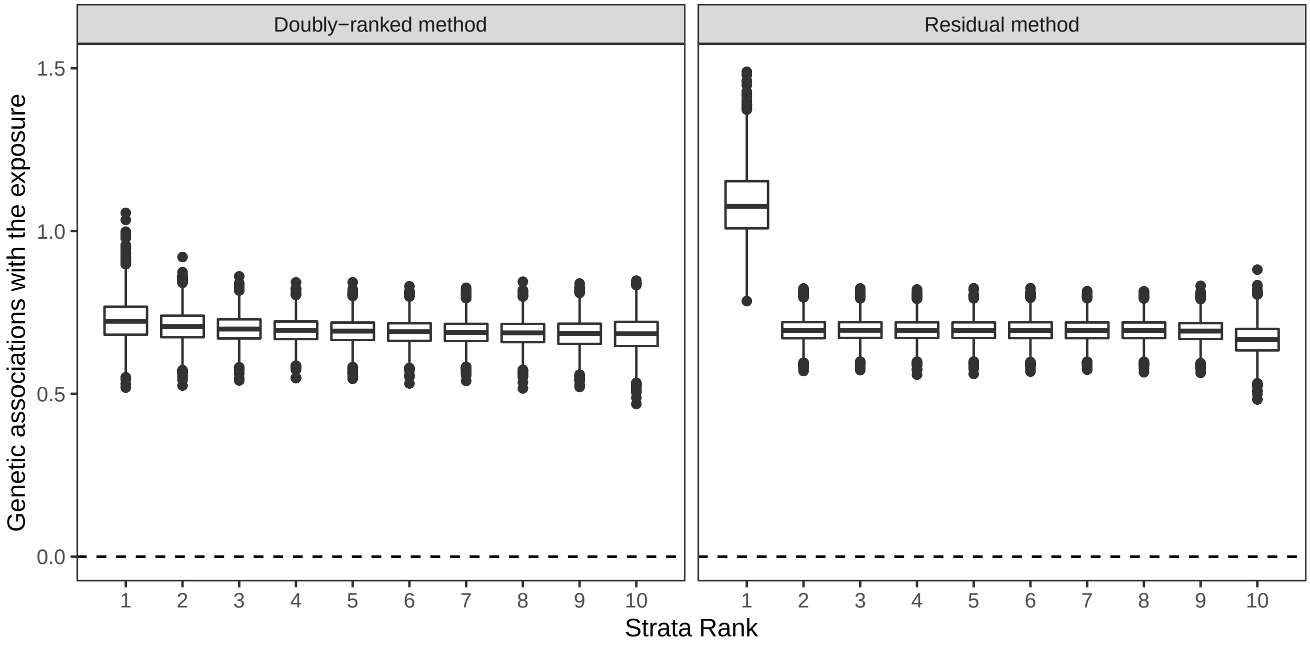

Fig B: Results of the doubly-ranked method and residual method for model B (nonlinearity and homogeneity). Boxplot results represent the estimates of genetic associations with the exposure within the 10 strata under 3000 simulations. Box indicates lower quartile, median, and upper quartile; error bars represent the minimal and maximal data point falling in the 1.5 interquartile range distance from the lower/upper quartile; estimates outside this range are plotted separately.

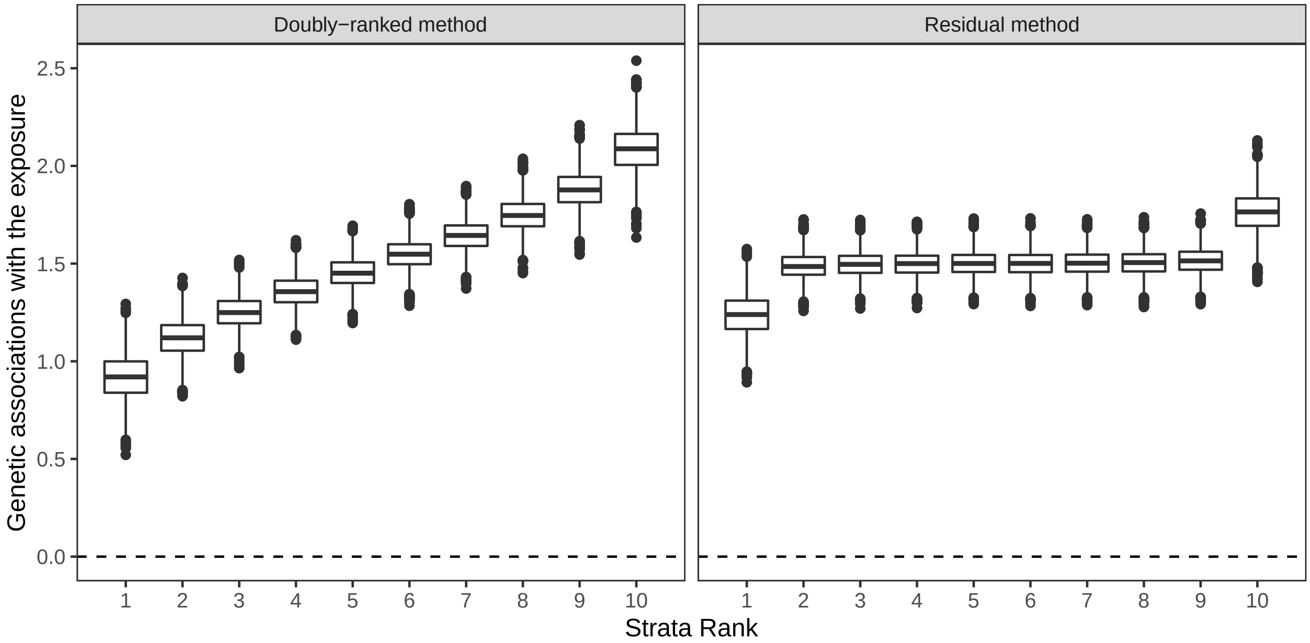

Fig C: Results of the doubly-ranked method and residual method for model C (linearity and heterogeneity). Boxplot results represent the estimates of genetic associations with the exposure within the 10 strata under 3000 simulations. Box indicates lower quartile, median, and upper quartile; error bars represent the minimal and maximal data point falling in the 1.5 interquartile range distance from the lower/upper quartile; estimates outside this range are plotted separately.

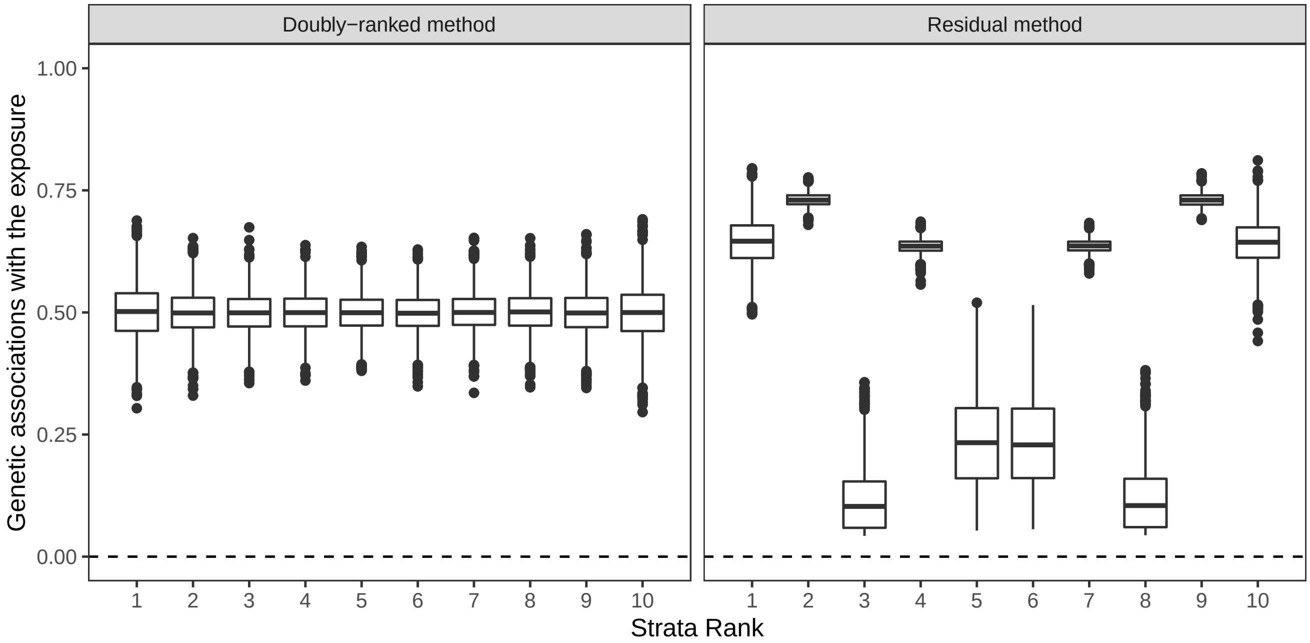

Fig D: Results of the doubly-ranked method and residual method for model D (coarsened exposures). Boxplot results represent the estimates of genetic associations with the exposure within the 10 strata under 3000 simulations. Box indicates lower quartile, median, and upper quartile; error bars represent the minimal and maximal data point falling in the 1.5 interquartile range distance from the lower/upper quartile; estimates outside this range are plotted separately.

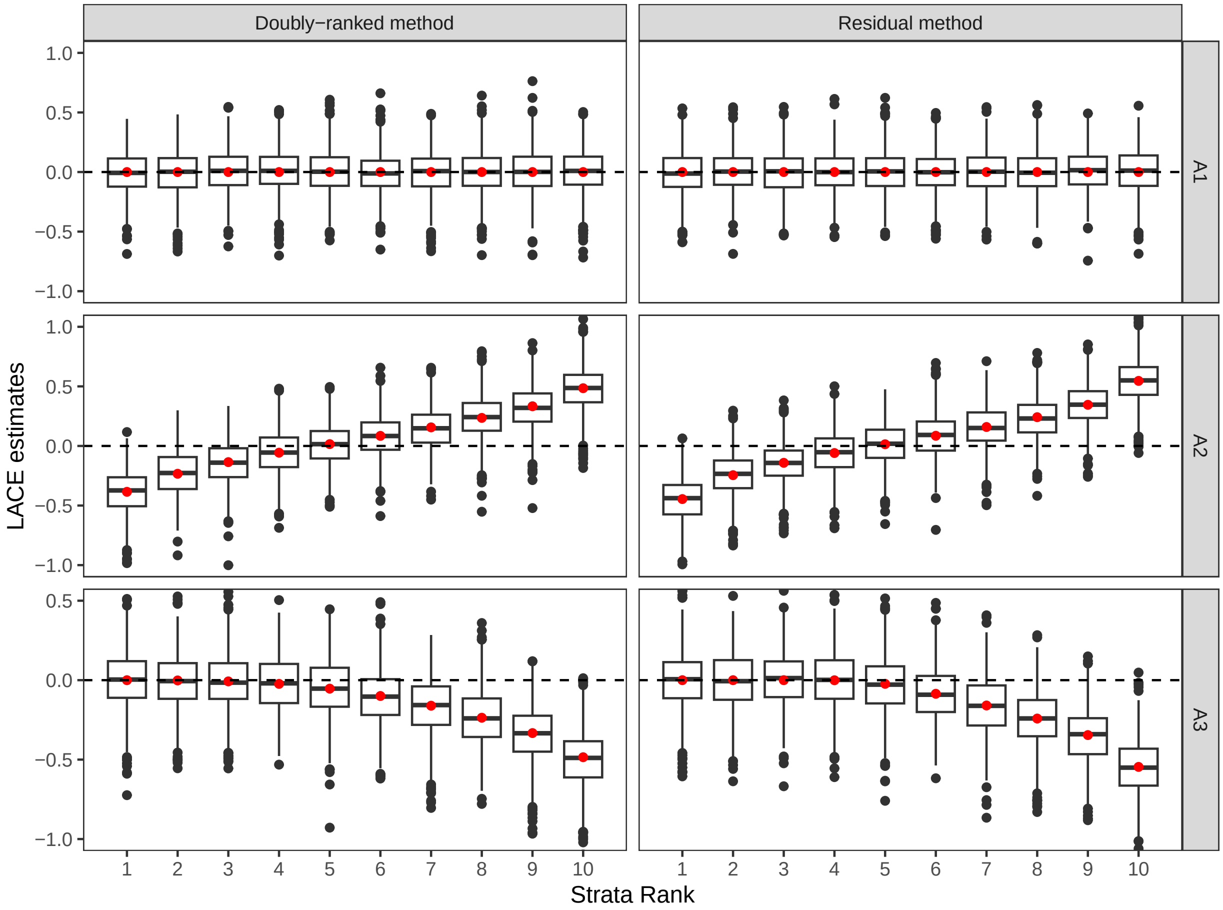

Fig E: Results of the doubly-ranked method and residual method for model A (linearity and homogeneity) with dichotomous instrument and three different causal relationship between the exposure and the outcome (denoted by A1, A2, A3). Boxplot results represent the LACE estimates within the 10 strata. Red points represent the target causal effects within strata. Box indicates lower quartile, median, and upper quartile; error bars represent the minimal and maximal data point falling in the 1.5 interquartile range distance from the lower/upper quartile; estimates outside this range are plotted separately.

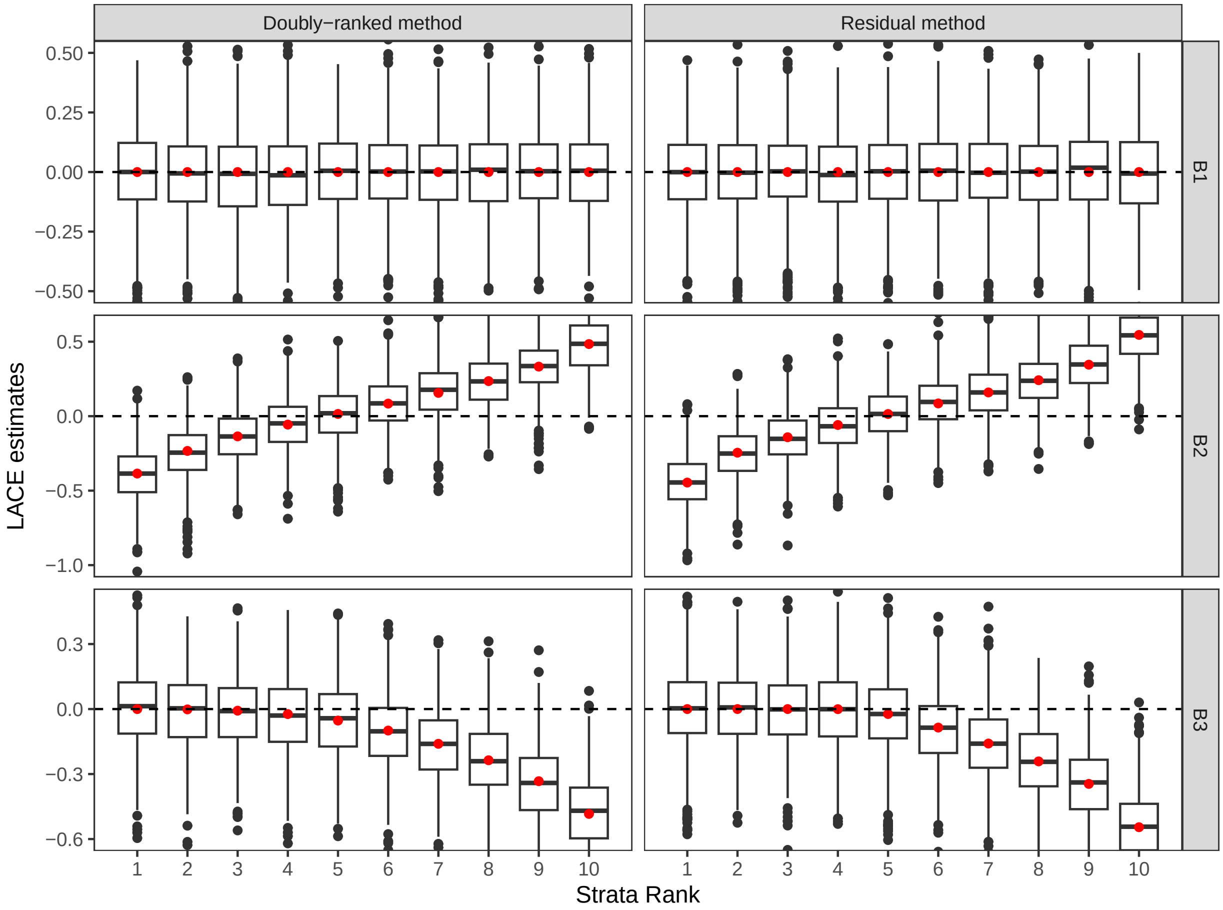

Fig F: Results of the doubly-ranked method and residual method for model B (nonlinearity and homogeneity) with dichotomous instrument and three different causal relationship between the exposure and the outcome (denoted by B1, B2, B3). Note that for a dichotomous instrument, model B degenerates to a linear and homogeneous model, and so the residual method performs well in this specific case. Boxplot results represent the LACE estimates within the 10 strata. Red points represent the target causal effects within strata. Box indicates lower quartile, median, and upper quartile; error bars represent the minimal and maximal data point falling in the 1.5 interquartile range distance from the lower/upper quartile; estimates outside this range are plotted separately.

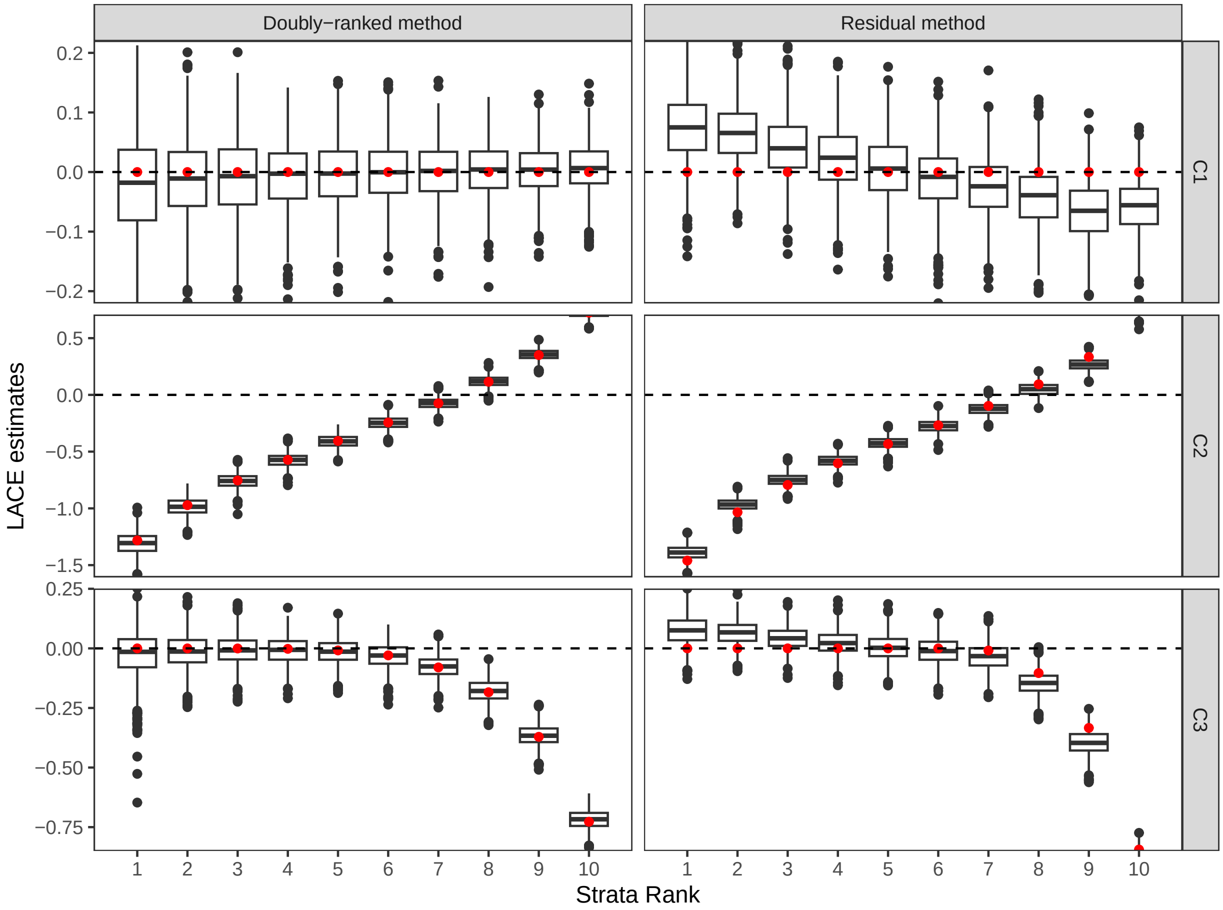

Fig G: Results of the doubly-ranked method and residual method for model C (linearity and heterogeneity) with dichotomous instrument and three different causal relationship between the exposure and the outcome (denoted by C1, C2, C3). Boxplot results represent the LACE estimates within the 10 strata. Red points represent the target causal effects within strata. Box indicates lower quartile, median, and upper quartile; error bars represent the minimal and maximal data point falling in the 1.5 interquartile range distance from the lower/upper quartile; estimates outside this range are plotted separately.

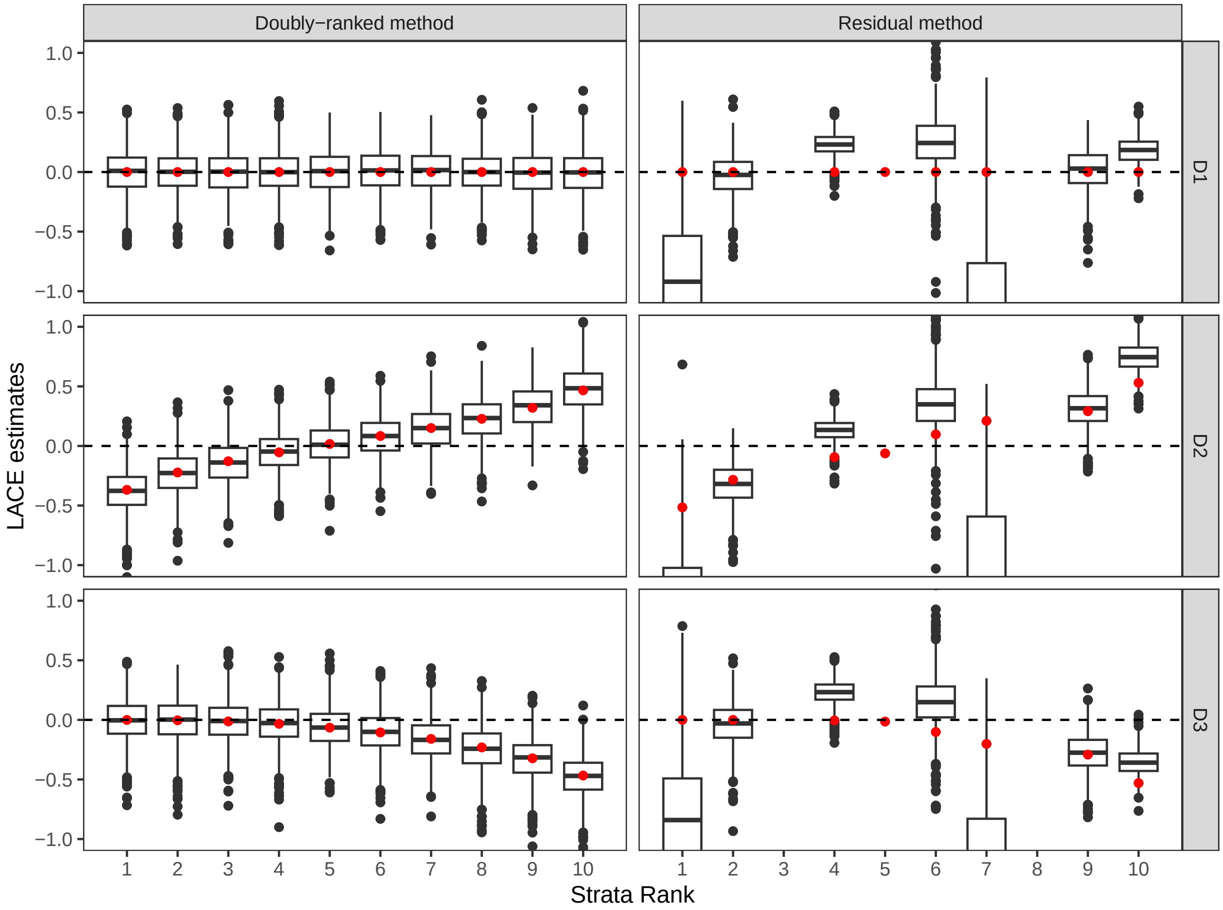

Fig H: Results of the doubly-ranked method and residual method for model D (coarsened exposures) with dichotomous instrument and three different causal relationship between the exposure and the outcome (denoted by D1, D2, D3). Boxplot results represent the LACE estimates within the 10 strata. Red points represent the target causal effects within strata. Estimates in the stratum where the instrument value is single are omitted. Box indicates lower quartile, median, and upper quartile; error bars represent the minimal and maximal data point falling in the 1.5 interquartile range distance from the lower/upper quartile; estimates outside this range are plotted separately.

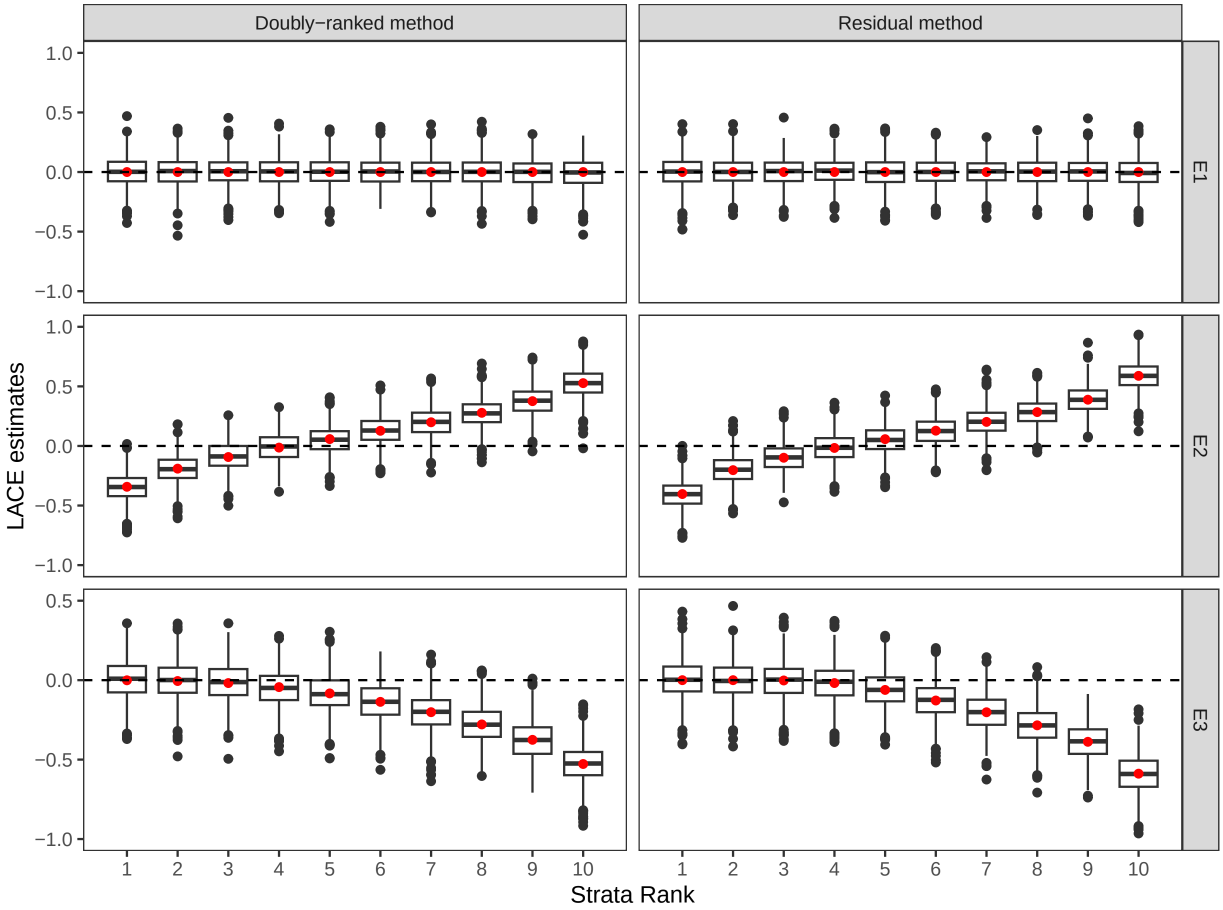

Fig I: Results of the doubly-ranked method and residual method for model E (homogeneity) with high-dimensional instruments and three different causal relationship between the exposure and the outcome (denoted by E1, E2, E3). Boxplot results represent the LACE estimates within the 10 strata. Red points represent the target causal effects within strata. Box indicates lower quartile, median, and upper quartile; error bars represent the minimal and maximal data point falling in the 1.5 interquartile range distance from the lower/upper quartile; estimates outside this range are plotted separately.

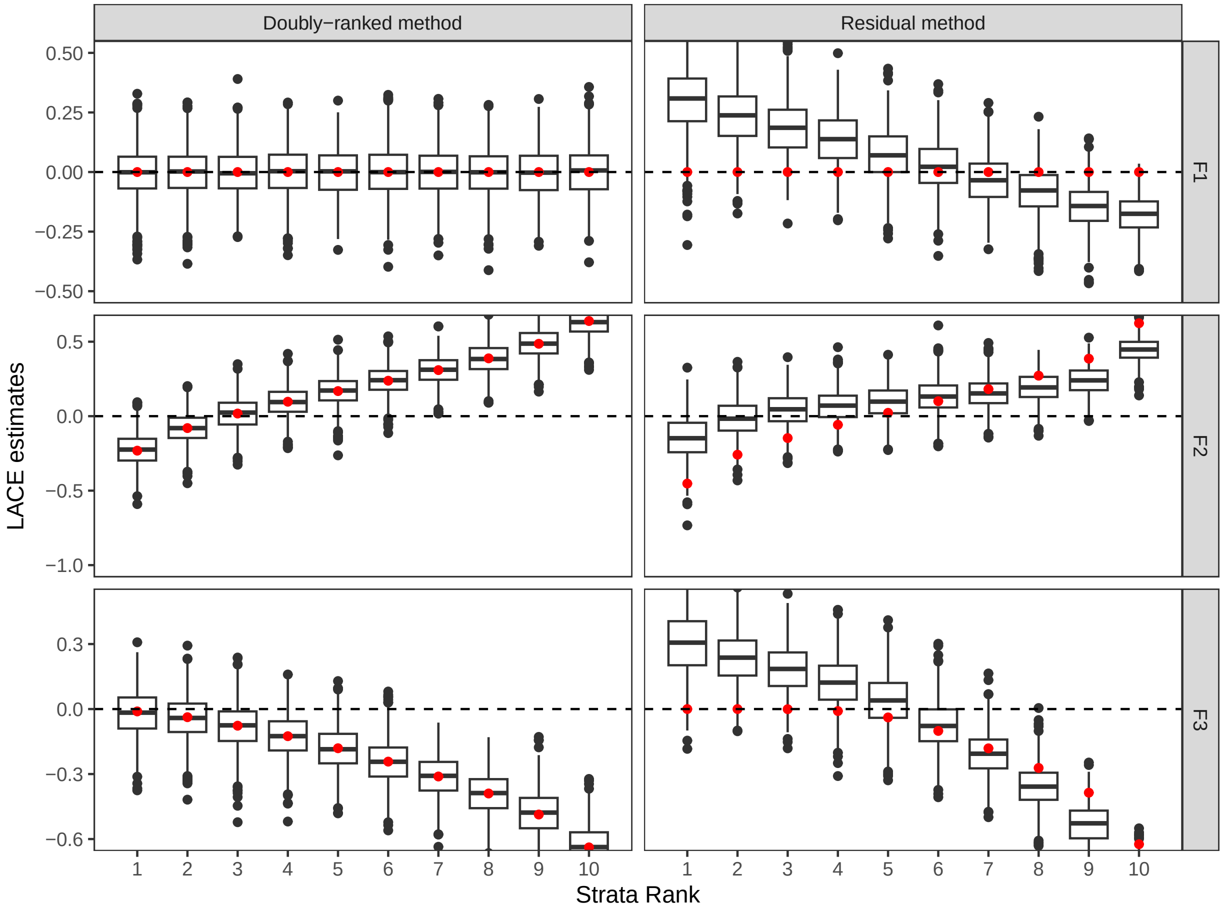

Fig J: Results of the doubly-ranked method and residual method for model F (interaction) with high-dimensional instruments and three different causal relationship between the exposure and the outcome (denoted by F1, F2, F3). Boxplot results represent the LACE estimates within the 10 strata. Red points represent the target causal effects within strata. Box indicates lower quartile, median, and upper quartile; error bars represent the minimal and maximal data point falling in the 1.5 interquartile range distance from the lower/upper quartile; estimates outside this range are plotted separately.

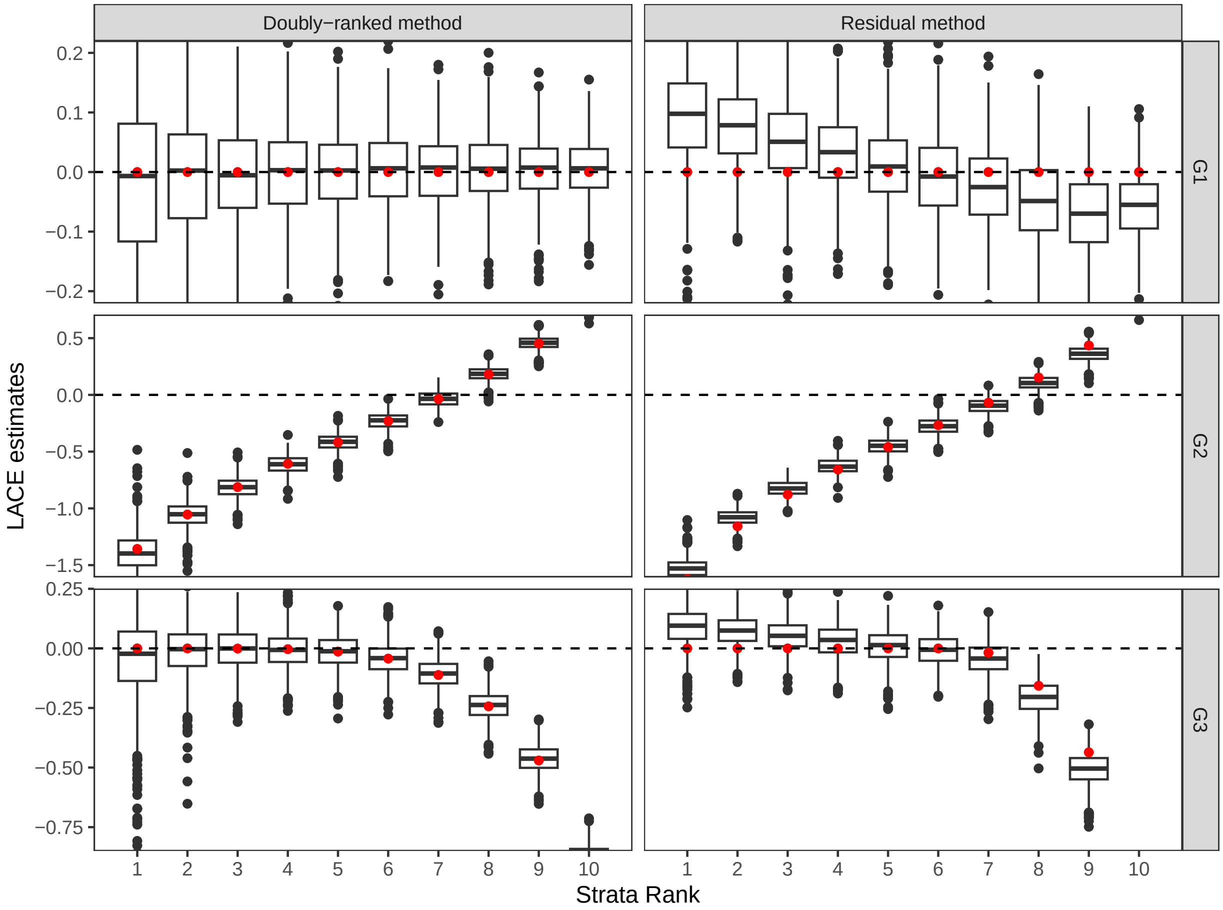

Fig K: Results of the doubly-ranked method and residual method for model G (heterogeneity) with high-dimensional instruments and three different causal relationship between the exposure and the outcome (denoted by G1, G2, G3). Boxplot results represent the LACE estimates within the 10 strata. Red points represent the target causal effects within strata. Box indicates lower quartile, median, and upper quartile; error bars represent the minimal and maximal data point falling in the 1.5 interquartile range distance from the lower/upper quartile; estimates outside this range are plotted separately.

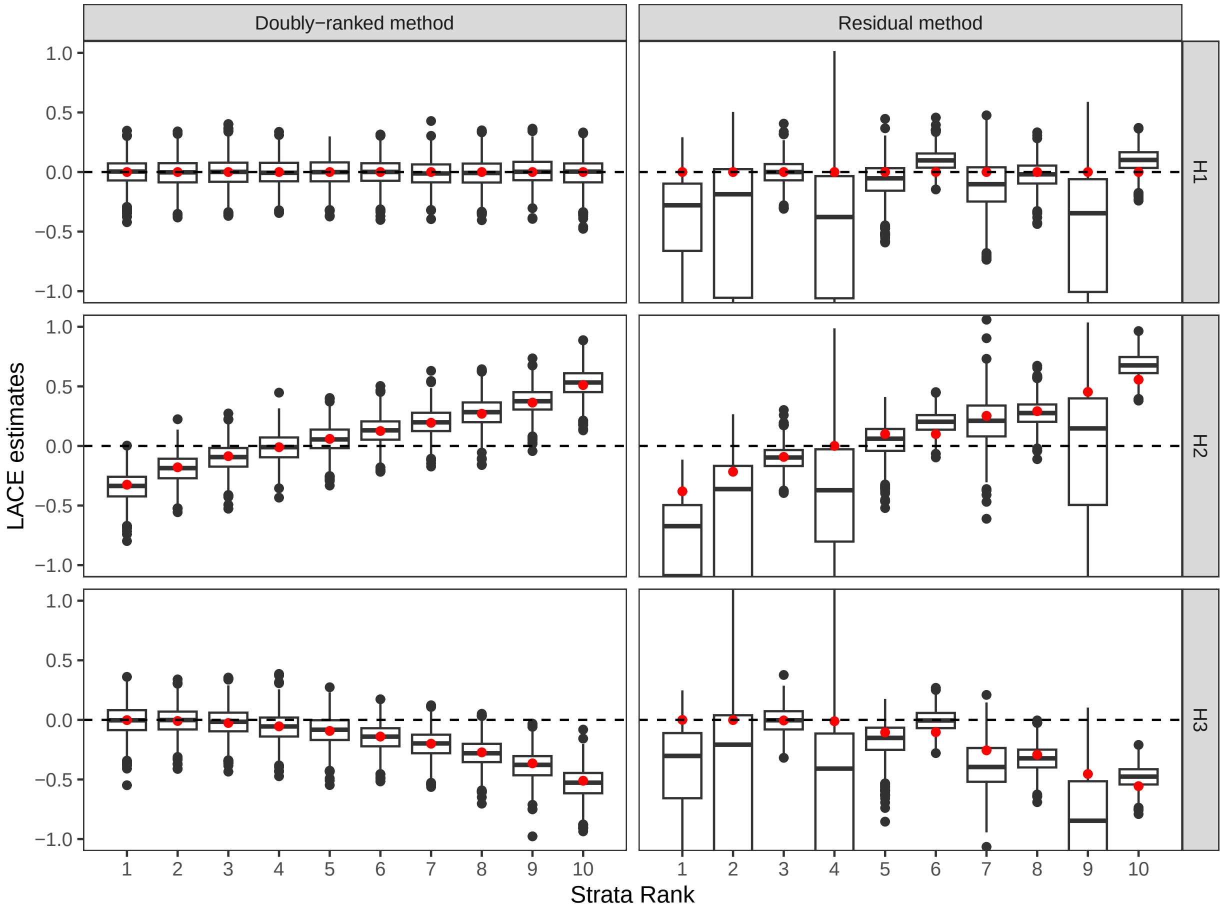

Fig L: Results of the doubly-ranked method and residual method for model H (coarsened exposures) with high-dimensional instruments and three different causal relationship between the exposure and the outcome (denoted by H1, H2, H3). Boxplot results represent the LACE estimates within the 10 strata. Red points represent the target causal effects within strata. Box indicates lower quartile, median, and upper quartile; error bars represent the minimal and maximal data point falling in the 1.5 interquartile range distance from the lower/upper quartile; estimates outside this range are plotted separately.

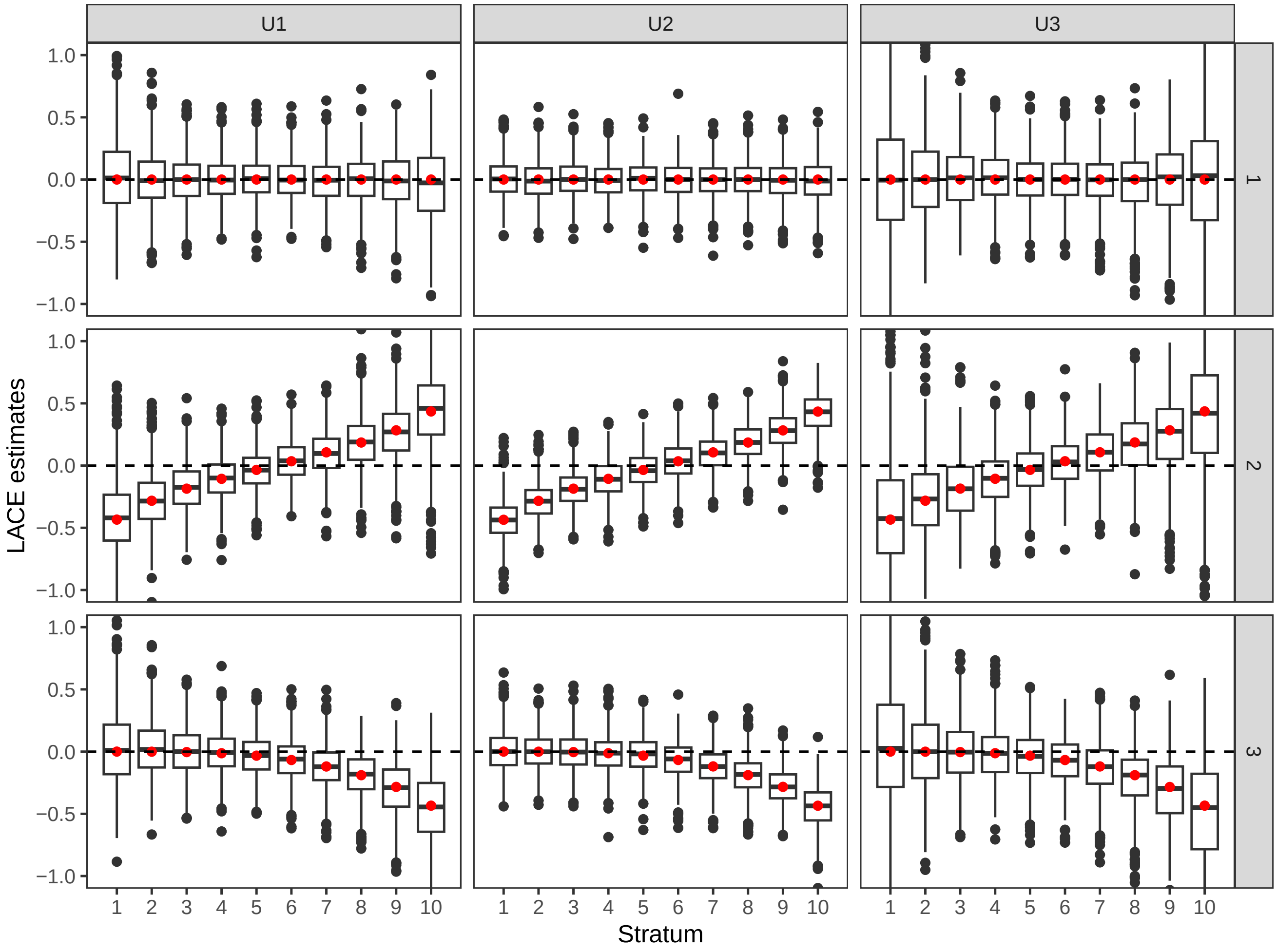

Fig M: Results of the doubly-ranked method for model A (linearity and homogeneity) with high-dimensional instruments and different exposure–outcome model (1,2,3) and confounder–outcome relationships (U1, U2, U3). Boxplot results represent the LACE estimates within the 10 strata. Red points represent the target causal effects within strata. Box indicates lower quartile, median, and upper quartile; error bars represent the minimal and maximal data point falling in the 1.5 interquartile range distance from the lower/upper quartile; estimates outside this range are plotted separately.

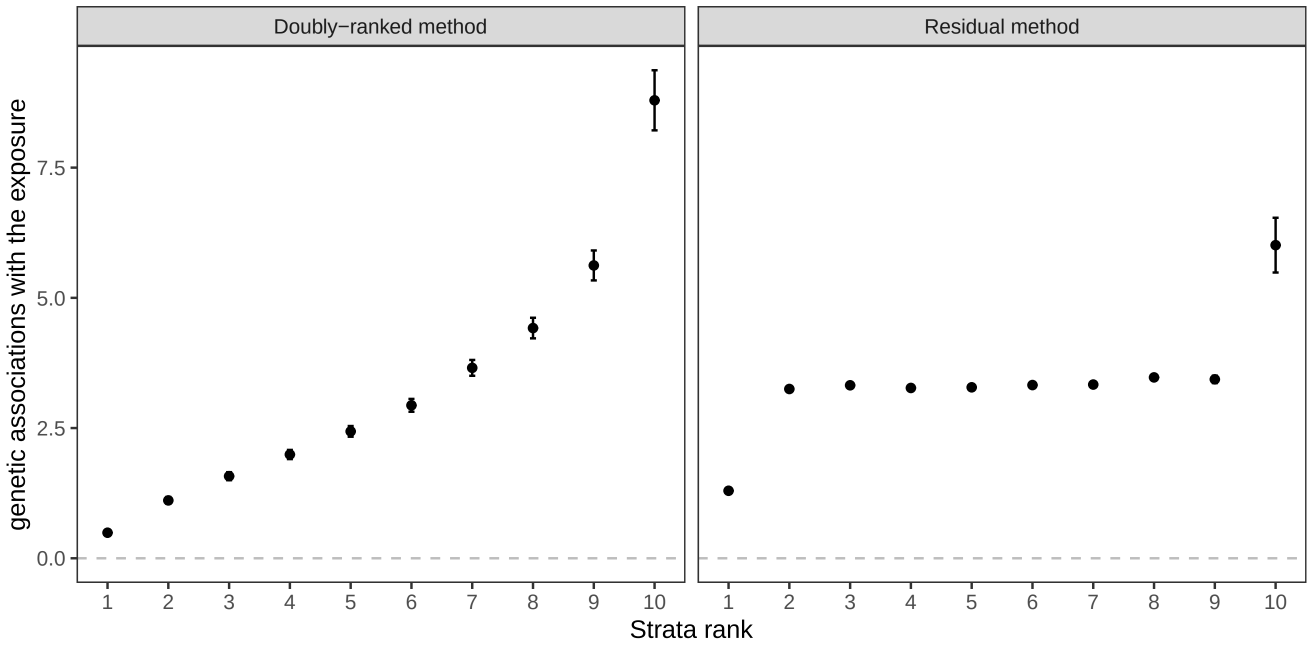

Fig N: The estimated genetic association with the exposure at each stratum for the residual and doubly-ranked stratification method with the real data of alcohol.

Text A: Exchangeability assessment for the coarsened exposure

We give guidance to choose the appropriate number of strata for coarsened exposures in the doubly-ranked stratification method. Let Z, X and U be the instrument, the (original) exposure, and error term (which incorporates the unmeasured confounding) respectively. Let the observed (coarsened) exposure be X^* and it satisfies the rank-preserving condition that $X_i > X_l$ for any $X^*_i > X^*_l$. For example, when exposure is coarsened by being rounded to the nearest integer value, $X_i > X_l$ for any $[X_i] > [X_l]$. Now for simplification assume the instrument Z takes K different values J times. We can build J strata via the doubly-ranked method according to the observed exposure X^*. Namely, the j-th strata (here we ignore the outcome information) is

$$\mathcal{S}_j = \bigcup_{k=1,\ldots,K} \{z_k; X^*_{(j)}|Z = z_k\} \tag{S1}$$

where $X^*_{(j)}|Z = z_k$ represents the j-th ranked exposure at the pre-strata of $Z = z_k$. When some observed exposures have the same value as a clump, we will randomly sort them. Due to the rank preservation property, the distribution of the error term at the k-th pre-strata (i.e. $Z = z_k$) and the j-th strata for the coarsened value of the exposure, denoted by $U^{\mathcal{S}_j}|Z = z_k$, can be expressed as a categorical random variable with the probability mass function

$$f(U^{\mathcal{S}_j} = U_{(r)}|Z = z_k) = N^{-1}_{j,k}\mathbf{1}\{X^*_{(r)} = X^*_{(j)}|Z = z_k\} \tag{S2}$$

where $N_{j,k}$ represents the size of the exposure clump in which the j-th ranked observed exposure of the k-th pre-stratum is located. That is,

$$N_{j,k} = \left|\{1 \le r \le J : X^*_{(r)} = X^*_{(j)}|Z = z_k\}\right| \tag{S3}$$

It is easy to know that the distribution of $U^{\mathcal{S}_j}|Z = z_k$ is determined by the two coefficients $a_{j,k}$ and $b_{j,k}$:

$$a_{j,k} = \max\{1 \le r \le J : X^*_{(r)} = X^*_{(j)}|Z = z_k\} \tag{S4}$$

$$b_{j,k} = \min\{1 \leq r \leq J : X^*_{(r)} = X^*_{(j)} | Z = z_k\} \tag{S5}$$

For the j-th stratum $\mathcal{S}_j$, the confounding should be approximately independent with the instrument (i.e., exchangeability) when the distribution of $U^{\mathcal{S}_j} | Z = z_k$ is uncorrelated with z_k. That is, $\{a_{j,k}, b_{j,k}\}_{k=1,2,...,K}$ has stabilized distribution over k. When exposure is precisely measured (i.e., the original exposure X), it is clear that $a_{j,k} = b_{j,k} = j$ and the confounding is exactly independent of the instrument. The stabilization can be checked in many ways. One heuristic method is the Gelman–Rubin convergence diagnostic, which evaluates whether multiple sample series (e.g. MCMC chain samples) have the converged distribution. In our analysis, we split $\{a_{j,k}\}_{k=1,2,...,K}$ (similar to $\{b_{j,k}\}_{k=1,2,...,K}$) into N^* equal parts, each of which is regarded as a chain with the length of $n^* = K/N^*$. We denote the elements of the t-th sample at the i-th chain by $x_{i,t}$, where $i \in \{1, 2, \ldots, N^*\}$ and $t \in \{1, 2, \ldots, n^*\}$. That is, $x_{i,t} = a_{j,n^*(i-1)+t}$. The between-chain variance estimate is

$$B = \frac{n^*}{N^* - 1} \sum_{i=1}^{N^*} \left(\bar{x}_i - \frac{1}{N^*} \sum_{i=1}^{N^*} \bar{x}_i \right)^2 \tag{S6}$$

where the mean for the i-th chain is $\bar{x}_i = \frac{1}{n^*} \sum_{t=1}^{n^*} x_{i,t}$. The within-chain variance is

$$W = \frac{1}{N^*} \sum_{i=1}^{N^*} s_i^2 \tag{S7}$$

where the variance for the i-th chain is $s_i^2 = \frac{1}{n^*-1} \sum_{t=1}^{n^*} (x_{i,t} - \bar{x}_i)^2$. The variance estimator is then

$$\hat{V} = \frac{n^* - 1}{n^*} W + \frac{1}{n^*} B \tag{S8}$$

Finally, the Gelman–Rubin statistic is

$$\hat{R} = \sqrt{\frac{\hat{V}}{W}} \tag{S9}$$

The stabilized samples of the clump coefficients will indicate that all chains are converging, then lead to $\hat{R}$ close to 1. In practice, we suggest splitting the samples into two halves (i.e., $N^* = 2$) and using the heuristic threshold 1.01 or 1.02 that are commonly used in practice. We recommend to choose the number of stratum in the doubly-ranked method such that all the strata have satisfied

a stabilization pattern of $\{a_{j,k}, b_{j,k}\}_{k=1,2,...,K}$. All the strata in the simulation and real studies of the main text with coarsened exposures have the Gelman–Rubin statistic lower than 1.02. Figure O gives one example of the assessment.

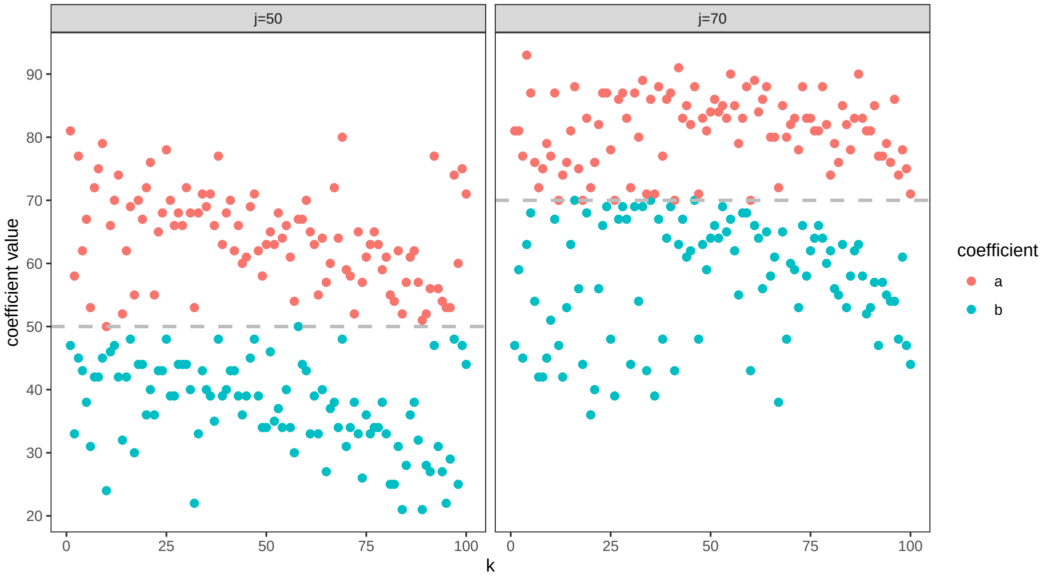

Fig O: One example of the exchangeability assessment for the 50th and 70th stratum in a simulation with the doubly-ranked method. The exposure is coarsened and the sample size is 10000 in which 100 strata were created. The coefficients $\{a_{j,k}, b_{j,k}\}_{k=1,2,...,100}$ are plotted against the stratum rank k. Left: the Gelman–Rubin statistics are 1.109 (for $a_{j,k}$) and 1.150 (for $b_{j,k}$) and the correlation of the confounder and the instrument is -0.135 (strong evidence exchangeability is violated). Right: the Gelman–Rubin statistics are 0.999 (for $a_{j,k}$) and 1.002 (for $b_{j,k}$) and the correlation of the confounder and the instrument is 0.025 (no strong evidence exchangeability is violated).

Text B: Additional simulation scenarios

We also consider two additional types of instruments in the simulation: dichotomous instruments and high-dimensional instruments. For the dichotomous instruments, the instrument-exposure models are the same as before (Models A to D), but the dichotomous instrument is defined as $Z \sim B(1, 0.3)$. Note that for dichotomous instruments, Model B degenerates to a linearity model. For the high-dimensional instruments, we consider four models with three dichotomous instruments, where $G_j \overset{\text{i.i.d}}{\sim} B(1, 0.3)$ for $j = 1, 2, 3$:

E. Linearity and homogeneity: $X = 0.2G_1 + 0.4G_2 + 0.6G_3 + U + \epsilon_X$

F. Interaction: $X = 0.1G_1 + 2.0G_2G_3 + U + \epsilon_X$

G. Heterogeneity: $X = -10 + (1.5 + 0.5U)(5 + 0.2G_1 + 0.4G_2 + 0.6G_3) + U + \epsilon_X$

H. As scenario E, but the exposure is coarsened by being rounded to the nearest integer value.

In model E, all effects are homogeneous. In model F, genetic interaction exists, which is a special example of heterogeneity. In model G, the genetic effects are heterogeneous and modified by the confounder U. In model H, the exposure is coarsened, making the model non-homogeneous for the coarsened values. When performing stratification with multiple instruments, a weighted gene score is constructed using weights obtained by fitting the regression of the exposure on the high-dimensional instruments. The results are presented in Supplementary Figures E to H for the dichotomous instrument and Supplementary Figures I to L for the independent instruments.

To assess the performance of the doubly-ranked stratification with more complex confounding situations, we conducted a simulation study using a continuous instrument under model A with three different confounder-outcome relationships:

U1. $Y = h(X) + U^2 + \epsilon_Y$

U2. $Y = h(X) + |U| + \epsilon_Y$

U3. $Y = h(X) + U + \epsilon_X^2 + 2U\epsilon_X + \epsilon_Y$

where $h(x)$ are the causal relationships in the exposure–outcome models 1-3. In case U1, the confounder U has a quadratic effect on the outcome. In case U2, the confounder U has a non-polynomial effect on the outcome. In case U3, the exposure-outcome confounders consist of U and ϵ_X, but their effects are different on the exposure and outcome, making it difficult to merge them into a single confounder. We consider the nine scenarios, comprising all combinations of causal relationships and confounder-outcome relationships, and simulated 1000 datasets per scenario. Results are shown in Supplementary Figure M and Supplementary Table A.

We conducted a simulation study to compare the performance of the stratification method and the PolyMR method in testing the linearity assumption of the exposure-outcome model. PolyMR evaluates the linearity assumption by performing a likelihood ratio test that compares the control regression model to the linear model (with only the first-degree exposure term). The stratification method evaluates the linearity assumption by testing the heterogeneity of the stratum-specific estimates with the Cochran's Q style statistic

$$Q = \sum_{k=1}^{K} \frac{(\hat{\beta}_{Yk} - \hat{\theta}\,\hat{\beta}_{Xk})^2}{\sigma_{Yk}^2 + \hat{\theta}^2 \sigma_{Xk}^2}. \tag{S10}$$

where K represents the number of strata, and $\hat{\beta}_{Xk}$ and $\hat{\beta}_{Yk}$ are the estimated instrument-exposure and instrument-outcome associations for each stratum. σ_{Xk} and σ_{Yk} are the corresponding standard errors, and $\hat{\theta}$ is the inverse-variance weighted average of the stratum-specific estimates. Under the null hypothesis of linearity, the statistic follows an approximate χ^2 distribution with $K-1$ degrees of freedom. We selected $K = 10$ as the number of strata, and we used causal model 1 as the null hypothesis scenario and causal model 2 as the alternative hypothesis scenario. Table 2 in the main text gives the results using 1000 simulations with different instrument–exposure models.

Text C: Mathematical details for the causal effect of each stratum

We give the details of the target causal effect in each stratum used for the calculation of MSE and coverage rate in the main text. For convenience, assume the structural equation (for a stratum) is

$$Y = h(X) + U + \epsilon_Y \tag{S11}$$

where X, Y, U and ϵ_Y are the exposure, the outcome, the unmeasured confounding and the exogenous variable respectively. Such an equation has satisfied the IV exclusion restriction assumption. We assume the effect shape $h(\cdot)$ is a differentiable function. Assume the IV assumptions (relevance, exchangeability, and exclusion restriction) for each stratum are satisfied. The MR will produce the estimator $\hat{\beta}$ with the form

$$\hat{\beta} = \frac{\hat{\theta}}{\hat{\alpha}} \tag{S12}$$

where $\hat{\theta}$ and $\hat{\alpha}$ are obtained from the regression (in each stratum)

$$X = \alpha_0 + \alpha Z + v_X \tag{S13}$$

$$Y = \theta_0 + \theta Z + v_Y \tag{S14}$$

where Z is the instrument and v_X, v_Y are the error terms. The MR estimator (S12) can also be derived with the same form by 2SLS and g-estimation. Let the exposure range be $[L, T]$ (w.l.o.g L and T can be negative and positve infinity). Now reconsider the equation (S11), which can be expressed as

$$\begin{aligned} Y =& h(X) + U + \epsilon_Y \\ =& h(L) + \int_L^X h'(x)dx + U + \epsilon_Y \\ =& h(L) + \int_L^T h'(x)X^*(x)dx + U + \epsilon_Y \end{aligned} \tag{S15}$$

where $X^*(x) := I\{X \geqslant x\}$ is defined as the value-varying exposure. Consider the value-varying regression

$$X^*(x) = \alpha_0^*(x) + \alpha^*(x)Z + \epsilon(x) \qquad x \in [L, T] \tag{S16}$$

with $\alpha^*(x) = \frac{cov(Z, X^*(x))}{var(Z)}$ such that $cov(Z, \epsilon(x)) = 0$ for any $x \in [L, T]$. The relevance condition ensures that $\alpha^*(x)$ cannot always equal to 0 over $[L, T]$. Hence, we can further express (S15) as

$$\begin{aligned} Y =& h(L) + \int_L^T h'(x)X^*(x)dx + U + \epsilon_Y \\ =& h(L) + \int_L^T h'(x)[\alpha_0^*(x) + \alpha^*(x)Z + \epsilon(x)]dx + U + \epsilon_Y \\ =& h(L) + \int_L^T h'(x)\alpha_0^*(x)dx + \int_L^T h'(x)\alpha^*(x)dx\, Z + \int_L^T h'(x)\epsilon(x)dx + U + \epsilon_Y \\ =& \theta_0^* + \theta^* Z + \epsilon^* \end{aligned} \tag{S17}$$

where

$$\theta_0^* := h(L) + \int_L^T h'(x)\alpha_0^*(x)dx$$

$$\theta^* := \int_L^T h'(x)\alpha^*(x)dx$$

$$\epsilon^* := \int_L^T h'(x)\epsilon(x)dx + U + \epsilon_Y$$

As $cov(Z, \int_L^T f(x)\epsilon(x)dx) = 0$ for general functions $f(x)$ including $h'(x)$ with regular conditions, $cov(Z, \epsilon^*) = 0$ due to the exchangeability condition. The slope estimator by fitting the regression (S14) will converge in probability as

$$\hat{\theta} \xrightarrow{p} \theta = \theta^* = \int_L^T h'(x)\alpha^*(x)dx \tag{S18}$$

The MR estimator will then converge in probability as

$$\hat{\beta} = \frac{\hat{\theta}}{\hat{\alpha}} \xrightarrow{p} \frac{\theta^*}{\alpha} = \int_L^T h'(x)\frac{\alpha^*(x)}{\alpha}dx \tag{S19}$$

The weight function of the target effect over $[L, T]$ is

$$
\begin{aligned}
W(x) :=& \frac{\alpha^*(x)}{\alpha} \\
=& \frac{cov(Z, X^*(x))}{cov(Z, X)} \\
=& \frac{\mathbb{E}(ZI\{X \geqslant x\}) - \mathbb{E}(Z)\mathbb{E}(I\{X \geqslant x\})}{\mathbb{E}(ZX) - \mathbb{E}(Z)\mathbb{E}(X)} \\
=& \frac{[\mathbb{E}(Z|X \geqslant x) - \mathbb{E}(Z)]P(X \geqslant x)}{\mathbb{E}(ZX) - \mathbb{E}(Z)\mathbb{E}(X)}
\end{aligned}
\tag{S20}
$$

The weight function can be replaced by the empirical estimates or expressed via Monte Carlo simulation for each stratum. That is, we derive the estimate as

$$
\widehat{W}(x^*) = \frac{[\widehat{\mathbb{E}}(Z|X \geqslant x^*) - \widehat{\mathbb{E}}(Z)]\widehat{P}(X \geqslant x^*)}{\widehat{\mathbb{E}}(ZX) - \widehat{\mathbb{E}}(Z)\widehat{\mathbb{E}}(X)} \tag{S21}
$$

for multiple exposure candidate values $x^* \in \mathcal{X}$. We then smooth $\left\{\left(x^*, \widehat{W}(x^*)\right)\right\}$ via smoothing methods like B-spline to obtain the estimated weight function $\widehat{W}(x)$. The target causal effect, β_T, is therefore

$$
\beta_T := \int_L^T h'(x)\widehat{W}(x)dx \tag{S22}
$$

which is the weighted integral of the derivative of the function relating the exposure to the outcome with the weight function estimated by the instrument and exposure values of individuals in each stratum. As $\widehat{W}(x^*) \xrightarrow{p} W(x^*)$ for any x^* as $n \to \infty$, $\beta_T \xrightarrow{p} \int_L^T h'(x)\frac{\alpha^*(x)}{\alpha}dx$. This means the MR estimator will converge to the target causal effect for each stratum. In other words, under the core IV assumptions, the MR estimator should be close to the target causal effect. As the relevance and exclusion restriction should hold in any stratum, the usage of the target causal effect is equivalent to evaluating the exchangeability for each stratum. One could also use other statistics to assess the performance of stratification, for example, using the sample correlations of the instrument and the confounders in each stratum. However, the target causal effect has the advantage of being more intuitive to visualise with MR estimators.
